# Cardiomyocyte ZKSCAN3 regulates remodeling following pressure‐overload

**DOI:** 10.14814/phy2.15686

**Published:** 2023-05-05

**Authors:** Xiaosen Ouyang, Sayan Bakshi, Gloria A. Benavides, Zhihuan Sun, Gerardo Hernandez‐Moreno, Helen E. Collins, Mariame S. Kane, Silvio Litovsky, Martin E. Young, John C. Chatham, Victor Darley‐Usmar, Adam R. Wende, Jianhua Zhang

**Affiliations:** ^1^ Department of Pathology University of Alabama at Birmingham Birmingham Alabama USA; ^2^ Department of Medicine University of Alabama at Birmingham Birmingham Alabama USA; ^3^ Birmingham VA Medical Center University of Alabama at Birmingham Birmingham Alabama USA; ^4^ Present address: Department of Materials Science and Engineering, Laboratory for Polymers & Healthcare Materials/Devices The University of Alabama at Birmingham (UAB) Birmingham AL USA; ^5^ Present address: Division of Environmental Medicine, Center for Cardiometabolic Science The University of Louisville Louisville KY USA; ^6^ Present address: Birmingham VA Health Care System (BVACS) AL USA

## Abstract

Autophagy is important for protein and organelle quality control. Growing evidence demonstrates that autophagy is tightly controlled by transcriptional mechanisms, including repression by zinc finger containing KRAB and SCAN domains 3 (ZKSCAN3). We hypothesize that cardiomyocyte‐specific ZKSCAN3 knockout (Z3K) disrupts autophagy activation and repression balance and exacerbates cardiac pressure‐overload‐induced remodeling following transverse aortic constriction (TAC). Indeed, Z3K mice had an enhanced mortality compared to control (Con) mice following TAC. Z3K‐TAC mice that survived exhibited a lower body weight compared to Z3K‐Sham. Although both Con and Z3K mice exhibited cardiac hypertrophy after TAC, Z3K mice exhibited TAC‐induced increase of left ventricular posterior wall thickness at end diastole (LVPWd). Conversely, Con‐TAC mice exhibited decreases in PWT%, fractional shortening (FS%), and ejection fraction (EF%). Autophagy genes (*Tfeb, Lc3b,* and *Ctsd*) were decreased by the loss of ZKSCAN3. TAC suppressed *Zkscan3, Tfeb, Lc3b*, and *Ctsd* in Con mice, but not in Z3K. The *Myh6/Myh7* ratio, which is related to cardiac remodeling, was decreased by the loss of ZKSCAN3. Although *Ppargc1a* mRNA and citrate synthase activities were decreased by TAC in both genotypes, mitochondrial electron transport chain activity did not change. Bi‐variant analyses show that while in Con‐Sham, the levels of autophagy and cardiac remodeling mRNAs form a strong correlation network, such was disrupted in Con‐TAC, Z3K‐Sham, and Z3K‐TAC. *Ppargc1a* also forms different links in Con‐sham, Con‐TAC, Z3K‐Sham, and Z3K‐TAC. We conclude that ZKSCAN3 in cardiomyocytes reprograms autophagy and cardiac remodeling gene transcription, and their relationships with mitochondrial activities in response to TAC‐induced pressure overload.

## Graphical abstract

Our study demonstrated that ZKSCAN3 level is significantly decreased, along with TFEB, in response to pressure overload. We then generated cardiomyocyte‐specific ZKSCAN3 knockout (Z3K) mice, and found that Z3K mice had an enhanced mortality compared to control (Con) mice following TAC, and those that survived exhibited a lower body weight. Significant changes of transcription landscapes were found in Z3K mice after sham surgery.

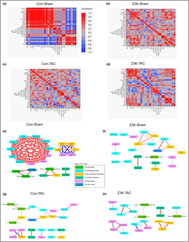

## 1 INTRODUCTION

Autophagy is a process of lysosomal‐mediated protein degradation that controls the accumulation of over‐produced, long‐lived, or damaged proteins and organelles (Zhang, 2013, 2015). Autophagy of the mitochondria (mitophagy) is essential for mitochondrial quality control (Hill et al., 2019; Kim et al., 2007; Redmann et al., 2014; Zhang, 2013). Impaired autophagy is associated with cardiac dysfunction (Gustafsson & Gottlieb, 2009; Lavandero et al., 2015; Nishida & Otsu, 2016; Schiattarella & Hill, 2016; Wang & Cui, 2017), enlarged mitochondria in the heart (Liang et al., 2020; Liang & Gustafsson, 2020), and insufficient cardiac protection during ischemia/reperfusion (Huang et al., 2010; Yitzhaki et al., 2009). Activation of autophagy by overexpression of Beclin has been shown to be cardioprotective in ischemia/reperfusion (Hamacher‐Brady et al., 2006) and AMPK activation by metformin or inhibition of histone deacetylase by suberoylanilide hydroxamic acid (SAHA) have been found to activate autophagy and elicit cardioprotection in diabetes and ischemia/reperfusion models (Xie et al., 2011, 2014).

The role of cardiac autophagy in response to hypertrophy is controversial. In response to cardiac hypertrophy there is an early adaptive increase in autophagy followed by a maladaptive reduction in autophagy (Lavandero et al., 2015; Rothermel & Hill, 2008; Wang & Cui, 2017). An example of detrimental effect of increasing autophagy has been demonstrated by that overexpression of Beclin‐1 accelerates the decline of cardiac function following pressure overload (Zhu et al., 2007) and inhibition of autophagy restores cardiac function or prevents its dysfunction (Cao et al., 2011; Shirakabe et al., 2016). On the other hand, pharmacological stimulation of autophagy has been shown to attenuate cardiac hypertrophy and heart failure in response to pressure overload (Shirakabe et al., 2016; Wang & Cui, 2017). This dichotomy emphasizes our limited understanding of the role of autophagy in cardiac pathophysiology. Maladaptive remodeling can also been promoted by mitochondrial dysfunction (Dai et al., 2001). It has been shown that cardiac‐specific deletion of acetyl CoA carboxylase 2 resulted in less increase of glycolysis and less decrease of fatty acid oxidation compared to control in response to TAC (Kolwicz et al., 2012).

Recently, several transcription factors have been identified to regulate autophagy (Fullgrabe et al., 2014), such as transcription factor EB (TFEB) and zinc‐finger protein with KRAB and SCAN domains 3 (ZKSCAN3) (Fullgrabe et al., 2014). TFEB has been shown to activate the expression of autophagy and lysosomal related genes (Fullgrabe et al., 2014; Lapierre et al., 2015). ZKSCAN3 has been shown to regulate TFEB target genes and is considered a master repressor of autophagy, including *Map1‐lc3* (Fullgrabe et al., 2014). In response to an autophagic stimulus TFEB is dephosphorylated and translocates to the nucleus; whereas ZKSCAN3 is phosphorylated and translocates out of the nucleus (Chauhan et al., 2013a). Together, the balance between ZKSCAN3 and TFEB regulates lysosomal biogenesis/autophagy, consistent with the concept that they work together in the transcriptional regulation of autophagy and lysosomal genes (Fullgrabe et al., 2014).

The central role of TFEB and ZKSCAN3 in regulating lysosomal biogenesis and autophagy raises the intriguing possibility that they could be more selective therapeutic targets for treatment of diseases linked to impaired lysosomal biogenesis or autophagic insufficiency. Consistent with this, a number of studies have demonstrated that increasing TFEB has beneficial effects on the heart subjected to oxidative or ischemic stress (Liang & Gustafsson, 2022). Both TFEB insufficiency and overexpression have been demonstrated to be detrimental to cardiac health, that its insufficiency promotes cardiac hypertrophy (Song et al., 2021), and its overexpression sensitizes the heart to chronic pressure overload (Wundersitz et al., 2022). Furthermore, the relationship of nuclear and mitochondrial communication has been demonstrated by the observation that TFEB has been shown to regulate PGC‐1α that controls gene expression for mitochondrial structure and function, and that mitochondrial dysfunction can affect the transcription landscape (Erlich et al., 2018; Kim et al., 2018; Ryan & Hoogenraad, 2007; Tian et al., 2019). Despite these important observations, the role of ZKSCAN3 in the heart is not known and the inter‐relationship between ZKSCAN3 and TFEB in the heart has not been studied. Additionally, whether ZKSCAN3 plays a role in modulating mitochondrial remodeling in response to pressure overload is unclear.

To this end, how ZKSCAN3 levels and heart functions are changed by pressure overload in control and ZKSCAN3 deficient mice have not been investigated. In the current study, we bred the ZKSCAN3^f/f^ mice with mice carrying cardiomyocyte‐specific cre and generated cardiomyocyte specific ZKSCAN3 knockout (Z3K) mouse to determine function of ZKSCAN3 in the heart, and its role in cardiac remodeling in response to pressure‐overload induced by transverse aortic constriction (TAC). We observed a role of ZKSCAN3 in mouse survival after pressure overload, a role of ZKSCAN3 in regulating the expression of autophagy and cardiac remodeling genes, and a reprogramming of the relationship between mitochondrial electron transport chain activities with mitochondrial biogenesis transcription factor *Ppagc1a*, and a dissociation of relationship between mitochondrial electron transport chain activities with mitochondrial mass.

## 2 METHODS

### 2.1 Mice

Mice were all in C57BL/6J background and kept on a 12‐h light/dark cycle with free access to food and water. All experiments were conducted in accordance with approved protocols by the University of Alabama at Birmingham, Institutional Animal Care and Use Committee (IACUC). ZKSCAN3 knockout mice were generated by breeding *Zkscan3* ^*f/f*^ mice (Ouyang et al., 2021) with αMHC‐cre mice (The Jackson laboratory). Experimental mice were *Zkscan3* ^*f/f*^ and αMHC‐cre positive (Z3K) while control mice were littermates *Zkscan3* ^*f/f*^ and αMHC‐cre negative (Con). Primers for genotyping *Zkscan3* are: Forward primer 1 (NDEL1): AGG CCA TGC CTT AAT GGG TGG, reverse primer 1 (NDEL2): TGA TGT CAA CAG CAC TGC CTT GG; and reverse primer 2 (SC1): GGT TTG GTT TTG CCT GGT GCA AAT G. For cre: MHCa‐Cre F: ATGACAGACAGATCCCTCCTATCTCC, and MHCa‐Cre R: CTCATCACTCGTTGCATCATCGAC. For non‐pressure overload studies, both males and females are used at indicated ages (Supplemental Information). For pressure overload studies, only males are used as described in the next section. To avoid circadian dependent changes, all mice were sacrificed between 6 am and 8 am. Left ventricles were used for RT‐PCR and mitochondrial bioenergetics analyses after being powdered and aliquoted.

### 2.2 TAC‐induced pressure overload

Aortic banding was performed on male mice at 10–11 weeks of age as previously described (Wende et al., 2017). Briefly, mice are anesthetized in an isoflurane induction chamber and then secured to a 37°C heating pad and maintained under sedation on a nose‐cone ventilator at 1.5–2.0% isoflurane. The fur is removed with depilatory cream and the skin disinfected with 10% povidone‐iodine solution. A horizontal skin incision ~0.5–1.0 cm in length is made at the level of the suprasternal notch. A small ~3‐mm longitudinal cut is made in the proximal portion of the sternum. The thyroid is retracted allowing for visualization of the aortic arch and a wire with a snare on the end is passed under the aorta between the origin of the right innominate and left common carotid arteries. A ligation clip applicator calibrated to a 30G needle is used to place a 1/1 titanium microclip (Horizon) next to the aortic arch, the sham surgery is identical, but no clip is placed. The muscle, sternum, and skin are sutured closed, the mice are given an injection of buprenorphine (0.05 mg/kg), and the mice are allowed to recover on a warming pad until they are fully awake.

### 2.3 Echocardiography

Echocardiography was performed the day after surgery to confirm that similar pressure gradients were acquired after TAC, as well as 8 weeks post‐surgery for full assessment of systolic function and cardiac changes as previously described (Wende et al., 2020). Briefly, mice were anesthetized in an isoflurane induction chamber and then secured to a 37°C heating pad and maintained under sedation on a nose‐cone ventilator at 1.5–2.0% isoflurane. Heart rates were monitored and maintained at 400–500 beats per minute. The fur was removed with a depilatory cream and limbs were taped onto the metal EKG leads with warmed echo gel. 30‐ or 12‐MHz probes (VisualSonics) were used to capture images and analyzed using the Vevo 2100 or 3100 program, respectively. M‐mode images were used to measure LV Interventricular septal thickness (IVS), left ventricle (LV) internal dimensions (LVID) and LV posterior wall thicknesses (LVPW) at diastole (;d) and systole (;s) at the level of papillary muscles. LV ejection fraction (EF), LV fractional shortening (FS), and LV posterior wall thickening (PWT) were calculated as previously described (Gao et al., 2011) Tissues were harvested 1 week after the second echocardiographic assessment, when animals were 19–20 weeks of age. All analyses were from m‐mode images.

### 2.4 Quantitative real‐time RT‐PCR

RNA was prepared from the powdered left ventricles using RNeasy Plus Mini Kit (Qiagen, Cat#: 74134). cDNA synthesis was performed using High‐Capacity cDNA Reverse Transcription Kit (Thermo Fisher Scientific, Cat#: 4368814). Quantitative real‐time PCR was performed with SYBR Green Mastermix (Thermo Fisher Scientific, Cat: 43‐643‐46) with the following conditions: 95°C, 5 min; and then 40 cycles at 95°C, 10 s; 60°C, 10 s; 72°C, 15 s. Results were normalized to the levels of β‐actin. Forward (F) and reverse (R) primer sequences are listed in Table 1.

**TABLE 1:** Primer sets for indicated gene expression.

	Forward	Reverse
Becn1	AGCCTCTGAAACTGGACACG	CTTCCTCCTGGGTCTCTCCT
Ctsd	CCGGTCTTTGACAACCTGAT	TCAGTGCCACCAAGCATTAG
Lamp1	CTGTCGAGTGGCAACTTCAG	GGATACAGTGGGGTTTGTGG
Lamp2a	TGGCTAATGGCTCAGCTTTC	ATGGGCACAAGGAAGTTGTC
Map1‐lc3	GTGGAAGATGTCCGGCTCAT	TGGTCAGGCACCAGGAACTT
Sqstm1/P62	CGAGTGGCTGTGCTGTTC	TGTCAGCTCCTCATCACTGG
Tfe3	CATCCTCGATCCTGACAGCT	TGTGGCCTGCAGTGATATTGG
Tfeb	GCGGACAGATTGACCTTCAG	CTCTCGCTGCTCCTCCTG
Wipi2	GCTGTTCGCCAACTTCAAC	TCTGTTCCAGCTTATCCACAGA
Zkscan3	CTGGATGACACACCTCCACA	TTTCTGAGCCCTGAGCTGAT
Actb	GACGGCCAGGTCATCACTAT	AAGGAAGGCTGGAAAAGAGC
Myh6	ACTGTGGTGCCTCGTTCC	TTCCGTTTTCAGTTTCCGC
Myh7	CATTCTCCTGCTGTTTCCTTAC	CATGGCTGAGCCTTGGAT
Nppa	ATGGGCTCCTTCTCCATCA	CCTGCTTCCTCAGTCTGCTC
Nppb	GGATCTCCTGAAGGTGCTGT	TTCTTTTGTGAGGCCTTGGT
Ppagc1a	TGATGTGAATGACTTGGATACAGACA	GCTCATTGTTGTACTGGTTGGATATG
Serca2a	GAAACTACCTGGAACAACCCG	CTTTTCCCCAACCTCAGTCA

### 2.5 Mitochondrial electron transport chain activities

Left ventricles were pulverized in liquid nitrogen and homogenized in MAS buffer (70 mM sucrose, 220 mM mannitol, 5 mM KH_2_PO_4_, 5 mM MgCl_2_, 1 mM EGTA, and 2 mM HEPES, pH 7.4), centrifuged at 200 *g* for 10 min at 4°C. Protein concentration was determined by DC Protein Assay (Bio‐Rad) with the supernatant. One microgram per well of each homogenate was loaded into Seahorse XF96 microplate. The plate was then centrifuged at 2000 *g* for 20 min at 4°C. To each well, cytochrome c and alamethicin were added as described (Acin‐Perez et al., 2020; Benavides et al., 2022). To measure complex activities, substrates were added: NADH for complex I, succinate and rotenone for complex II, duroquinol for complex III, and ascorbate with TMPD (N,N,N′,N′‐tetramethyl‐para‐phenylene‐diamine) for complex IV as described (Acin‐Perez et al., 2020; Benavides et al., 2022). Activities were terminated by respective complex inhibitors including rotenone for complex I, antimycin A for complexes II and III, and azide for complex IV as described (Acin‐Perez et al., 2020; Benavides et al., 2022).

### 2.6 Citrate synthase and LDHA activity assays

Biochemical assays were performed by incubating protein lysates with oxaloacetate, acetyl CoA, and DTNB for citrate synthase activity assays, and with NADH and Pyruvate for LDHA activity assays. The reaction product DTNB‐CoA was monitored at 412 nm and the decrease of NADH was measured with absorption at 340 nm. The changes of absorbance were used to calculate activities as nmol/min/mg protein (Acin‐Perez et al., 2020; Benavides et al., 2022; Van et al., 2022).

### 2.7 Statistical analyses

Statistical analyses were performed by Two‐Way ANOVA followed by post hoc Šidák test, or Student's *t*‐test as appropriate using GraphPad Prism7. Data are presented as mean ± SEM. Multiple correlation analysis was performed using JMP Pro 16 with non‐parametric Kendall's rank correlation, Prob>|t| < 0.05 was considered significant. Visualization of networks was performed using Cytoscape 3.8.2.

## 3 RESULTS

### 3.1 Role of ZKSCAN3 in cardiac remodeling in response to pressure overload

We generated cardiomyocyte specific *Zkscan3* knockout mice (Z3K) by breeding Zkscan3^f/f^ with MHC‐cre and as expected, Z3K hearts exhibited decreased *Zkscan3* mRNA (Supplemental Information, S3D, S6B, S7D, S8A, J). We measured food intake, activity, respiratory exchange ratio (RER), and VO_2_ for 24 h, followed by the mRNA and protein levels of key autophagy genes. We found no significant baseline functional phenotypic differences between control and Z3K groups. In addition, both Z3K heterozygous and homozygous knockout mice exhibited an increase in autophagy gene expression in response to fasting suggesting that ZKSCAN3 does not limit the transcriptional control in response to this stress (Supplemental Information S3K, S8B‐J). These findings are consistent with earlier reports that the young adult global ZKSCAN3 KO mice exhibited no differences from wildtype mice for the mRNA levels of multiple putative targets of ZKSCAN3 under fed or fasting condition in the brain, heart, or liver; and no difference in LC3II protein levels in MEFs before and after starvation (Pan et al., 2017). These results are surprising since ZKSCAN3 has been shown to be a master transcription repressor in cell types other than cardiomyocytes and raises the question about the functional significance of this protein for the heart (Chauhan et al., 2013b).

**FIGURE 1:**
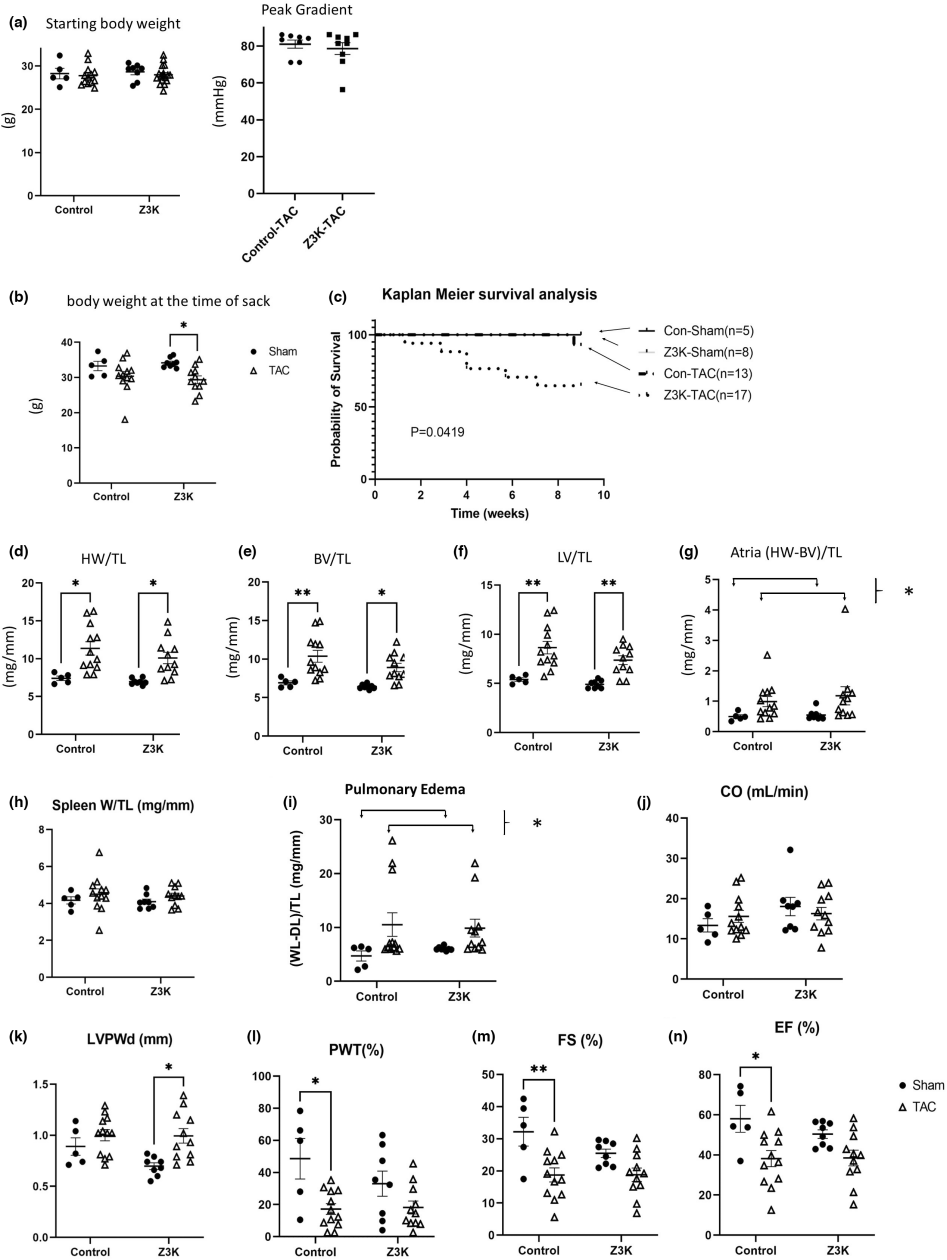
Whole body and cardiac phenotypes after TAC in wildtype control and *Zkscan3* cardiomyocyte specific knockout (Z3K) mice. (a) Starting body weight at 2 months of age were no different for all the mice used in this experiment. Peak gradients after TAC indicate comparable pressure overload in both Z3K mice and control (Con) mice. (b) Body weight at 19–20 weeks of age before sacrifice. There was a significant decrease of body weight for the Z3K mice after TAC (11 out of 17 survived) compared to after Sham. (c) Kaplan Meier survival analysis showed that Z3K mice have higher lethality after TAC compared to after Sham or control groups. Both control and Z3K mice exhibited increased heart weight/tibia length (d), biventricular volume/tibia length (e), left ventricular volume/tibia length (f), after TAC, but there was no genotype difference. (g) There was an increase of (heart weight‐biventricular volume)/tibia after TAC. (h) There was no significant spleen weight increase in either control or Z3K mice after TAC. (i) There was an increase of pulmonary edema after TAC. (j) There was no difference among the groups in cardiac output. (k) There was an increase of posterior wall thickness during diastolic phase after TAC for Z3K mice. (l). 100* [(PWs‐PWd)/PWd] (PWT%) was significantly decreased only in the wildtype control by TAC. (m, n) Only wildtype control mice exhibited a TAC effect in fractional shortening (FS%) and ejection fraction (EF%). *p < 0.05, **p < 0.01, ***p < 0.001, ****p < 0.0001. *n* = 5–17. Two‐way ANOVA and post hoc Šidák test. BW, body weight, HW, heart weight; TL, tibia length.

Autophagy has been shown to be suppressed starting from 5 days post‐transaortic constriction (TAC) (Shirakabe et al., 2016; Wang & Cui, 2017). To better understand the role of ZKSCAN3 in the heart we assessed the response to increased hemodynamic stress induced by TAC. In the first series of experiments 5–12 mice from each group were used for a measurement of key parameters (Table 2). We found no differences in body weight before surgery between genotypes, for either the surviving mice (data not shown) or all mice (Figure 1a). Compared to the Sham group, littermate control mice that survived TAC did not show a change in body weight, while Z3K mice that survived TAC had a body weight decrease compared to sham (Figure 1b). The survival curves showed that Z3K mice exhibit significantly higher mortality (Figure 1c).

**TABLE 2:** The numbers of mice in each group for Sham or TAC surgery.

	WT control‐sham	Z3K‐sham	WT control‐TAC	Z3K‐TAC
Survived	5	8	12	11
Dead	0	0	1	6

We performed tissue weight and cardiac function measurements for both genotypes with sham and TAC surgeries. In both genotypes following TAC there was an increase in heart weight/tibia length (Figure 1d), an increase of biventricular weight/tibia length (Figure 1e), and an increase in the left ventricle weight/tibia length (Figure 1f). There was no change in spleen weight/tibia length in any group of mice (Figure 1h), while an increase in atrial weight/tibia length (Figure 1g), and pulmonary edema as defined by (wet lung—dry lung) / tibia length was significant post TAC compared to sham (Figure 1i). For functional analyses, we found no difference in cardiac output among all groups (Figure 1j), an increase of the posterior wall thickness during diastolic phase (LVPWd) in Z3K group after TAC compared to the same genotype sham (Figure 1k), a decrease in PWT% in wildtype control group after TAC compared to the same genotype sham (Figure 1l), and decreases in FS% and EF% by TAC compared to sham in wildtype controls (Figure 1m,n). Table 3 list the two‐way ANOVA results of these analyses.

**TABLE 3:** Two‐way ANOVA analyses of body, tissue, and cardiac measurements.

	Genotype factor	TAC	Interaction
Start BW	ns	ns	ns
Stop BW	ns	**	ns
HW/TL	ns	***	ns
BV/TL	ns	****	ns
LV/TL	ns	****	ns
Atria (HW‐BV)/TL	ns	*	ns
Spleen W/TL	ns	ns	ns
Pulmonary Edema	ns	*	ns
CO	ns	ns	ns
LVPWd	ns	**	ns
PWT%	ns	***	ns
EF%	ns	**	ns
FS%	ns	***	ns

* <0.05

** <0.01

*** <0.001

**** <0.0001.

### 3.2 Role of ZKSCAN3 in modulating mitochondrial function in mice with sham and TAC surgery

**FIGURE 2:**
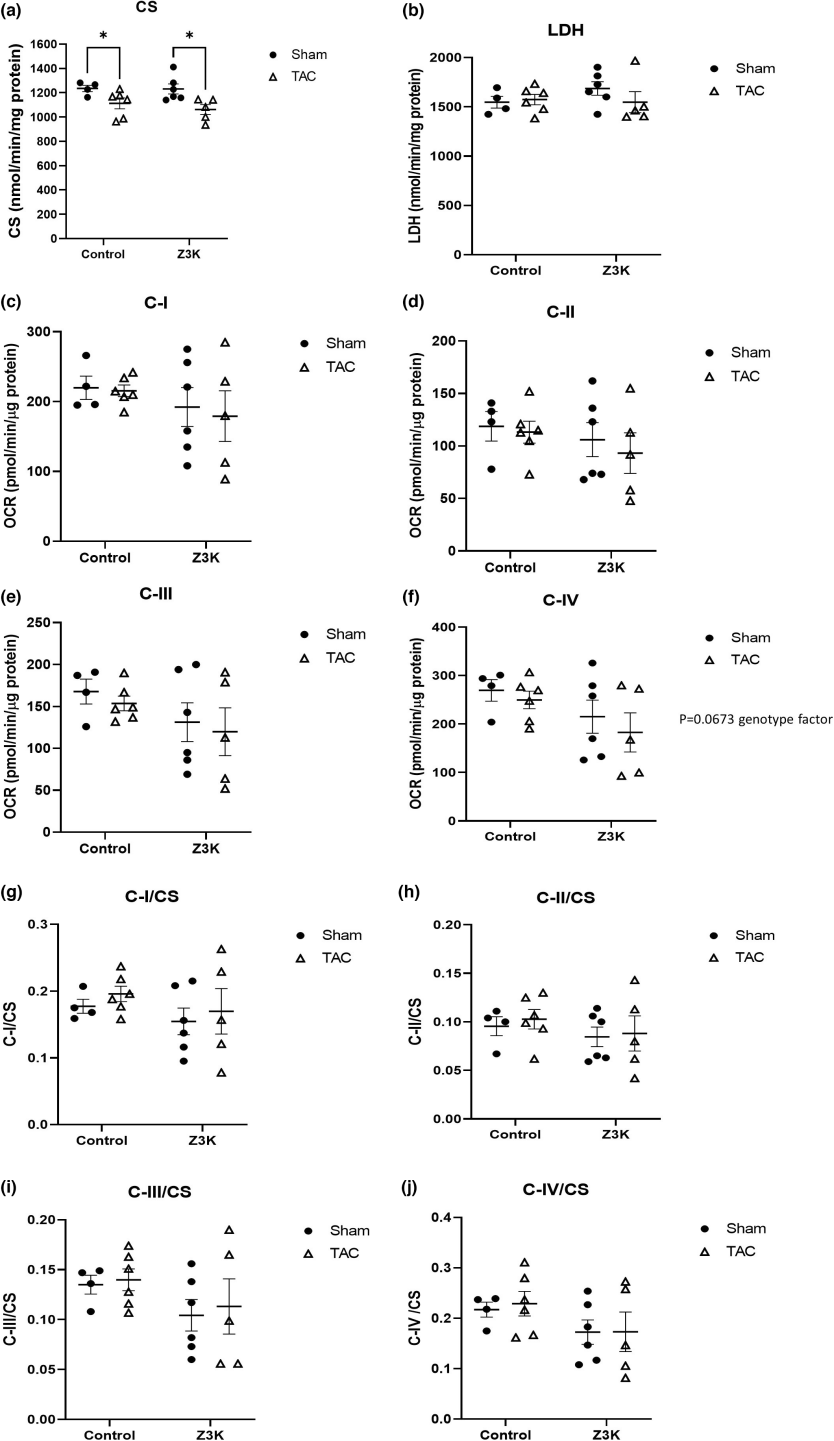
Citrate synthase, LDHA and mitochondrial complex I‐IV activities in wildtype control and Z3K mice after either sham or TAC. (a) Citrate synthase activities were decreased by TAC both in wildtype control and in Z3K mice. (b) No change in LDH activities by TAC or by ZKSCAN3 KO. (c–f) Mitochondrial complex I‐IV substrate linked activities were unchanged by TAC or by ZKSCAN3 KO. (g–j). Mitochondrial complex I‐IV activities normalized to citrate synthase activities. *p < 0.05, *n* = 4–6. Two‐way ANOVA and post hoc Šidák test.

Blinded electron microscopic analyses revealed no significant genotype differences for mitochondrial or lipid droplet numbers in the heart after 24 h fasting (Figure S5). To determine the effects of ZKSCAN3 cardiomyocyte specific KO and TAC on metabolism related to mitochondrial function and glycolysis, we performed citrate synthase (a mitochondrial matrix enzyme) and lactate dehydrogenase (LDH, a cytosolic glycolytic enzyme) activity measurements. As shown in Figure 2a, citrate synthase activities were similar between control and ZKSCAN3 KO mice in sham, while there was a significant decreased post‐TAC in both the control and the Z3K mice. LDH activities were similar in all 4 groups of mice (Figure 2b). No significant changes were evident in complexes I, II, III or IV substrate linked oxygen consumption rates (Figure 2c–f). Furthermore, there was no difference among the relative complexes I, II, III or IV substrate linked oxygen consumption rates normalized to the same amount of mitochondrial mass (as represented by citrate synthase activities) (Figure 2g–j). Interestingly, the variability in complex I, II, III or IV activities was larger in Z3K mice compared to control mice in both sham and TAC groups.

### 3.3 Role of ZKSCAN3 in transcriptional regulation of autophagy related genes

**FIGURE 3:**
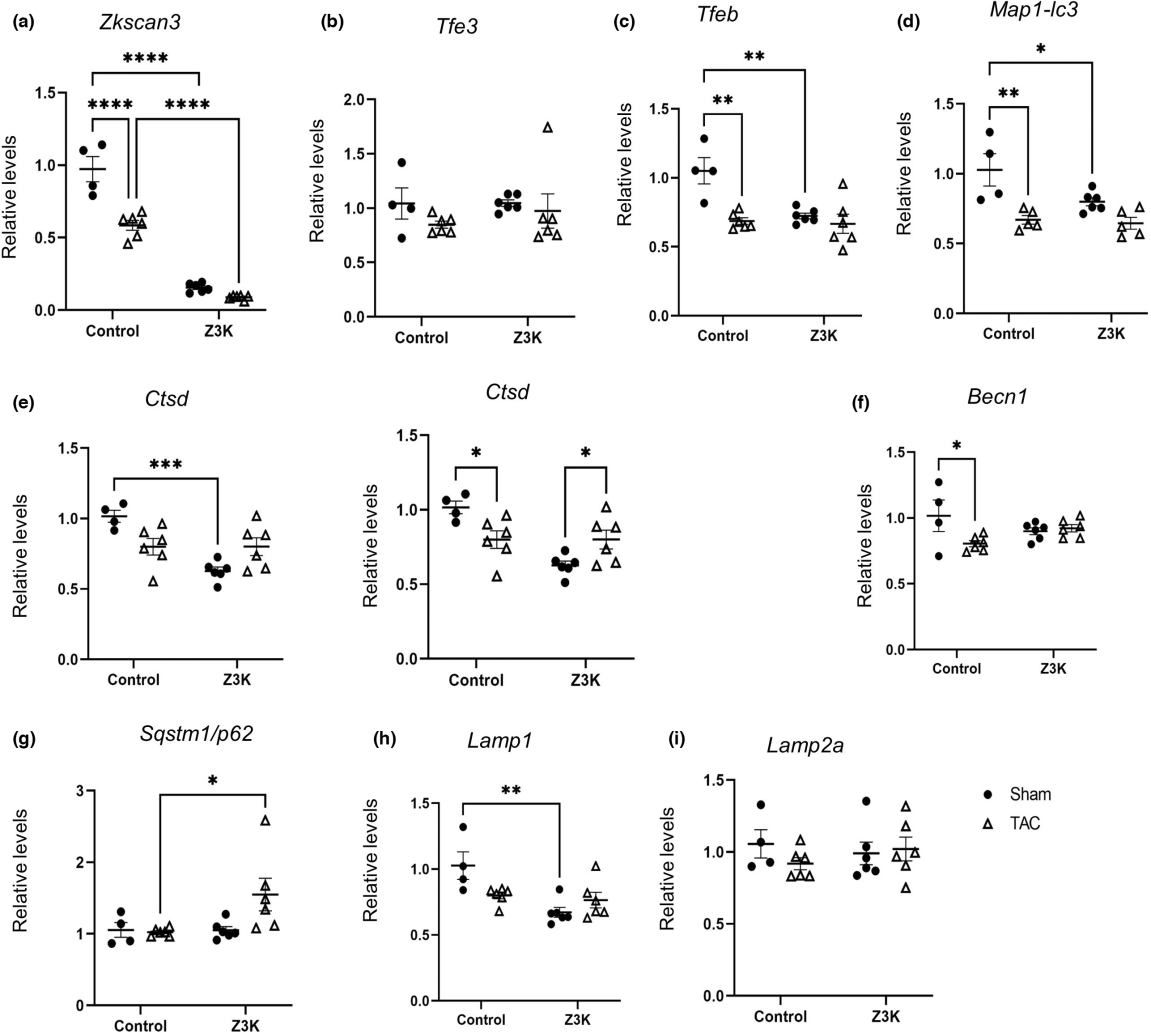
Reprogramming of autophagy gene expression by TAC in wildtype control and Z3K mice. We performed RT‐PCR analyses of autophagy‐lysosome pathway related genes in wildtype control and Z3K mice after either sham surgery or TAC. (a) There was a significant decrease of *Zkscan3* mRNA in TAC compared to Sham in the control mice, while Z3K mice exhibited even lower mRNA in both Sham and TAC. (b) *Tfe3* mRNA was changed neither by ZKSCAN3 knockout or by TAC. *Tfeb* (c), and *Map1‐lc3* (d) mRNAs were decreased in control mice by TAC to similar extent as by ZKSCAN3 knockout. *Ctsd* (e) mRNA was lower in Z3K sham compared to wildtype control sham. Its level was decreased in wildtype control and increased in Z3K by TAC. (f) *Becn1* mRNA was decreased in control by TAC. (g) *Sqstm1/p62* mRNA was higher in Z3K mice compared to wildtype control after TAC. (h) *Lamp1* mRNA was lower in Z3K mice compared to wildtype control in Sham groups. (i) No difference in *Lamp2* mRNA among the groups. *p < 0.05, **p < 0.01, ***p < 0.001, ****p < 0.0001. *n* = 4–6. Two‐way ANOVA and post hoc Šidák test.

To determine whether cardiomyocyte specific knockout of ZKSCAN3 led to alterations in gene expression related to the autophagy lysosome pathway in sham or TAC mice, we performed quantitative real time RT‐PCR (Table 4). As expected, *Zkscan3* mRNA was markedly decreased in Z3K hearts (Figure 3a). Interestingly, in control hearts, TAC decreased *Zkscan3* expression (Figure 3a). Although decreased *Zkscan3* expression might be expected to increase autophagy gene expression, the expression of *Tfeb, Map1‐lc3b, Ctsd* and *Becn1* mRNA were all decreased by TAC (Figure 3c–f). *Tfeb, Map1‐lc3b* and *Ctsd* were also significantly decreased in the Z3K sham group compared to wildtype control shams, consistent with an adaptive response of *Tfeb* to the loss of *Zkscan3*, and associated with a decrease in *Tfeb* target gene expression. TAC in Z3K mice did not result in a further decrease in these autophagy genes. In contrary, *Ctsd* was increased by TAC in Z3K mice (Figure 3e). *Sqstm1/p62* was similar between control and Z3K mice in sham conditions, but it was increased by TAC in Z3K but not control mice (Figure 3g). *Lamp1* mRNA was decreased in Z3K compared to control in sham condition, but with no further changes with TAC (Figure 3h). There was no difference in *Tfe3* and *Lamp2a* mRNA among the groups (Figure 3b,i).

**TABLE 4:** Two‐way ANOVA analyses of levels of autophagy lysosomal pathway related mRNAs.

	Genotype factor	TAC	Interaction
*Becn1*	ns	ns	*
*Ctsd*	**	Trends opposite between genotypes	**
*Lamp1*	**	Trends opposite between genotypes	**
*Lamp2a*	ns	ns	ns
*Map1‐lc3*	*	***	ns
*Sqstm1/P62*	ns	ns	ns
*Tfe3*	ns	ns	ns
*Tfeb*	**	**	*
*Zkscan3*	****	****	***

* <0.05

** <0.01

*** <0.001

**** <0.0001.

### 3.4 Role of ZKSCAN3 in the transcriptional response of the heart to TAC

**FIGURE 4:**
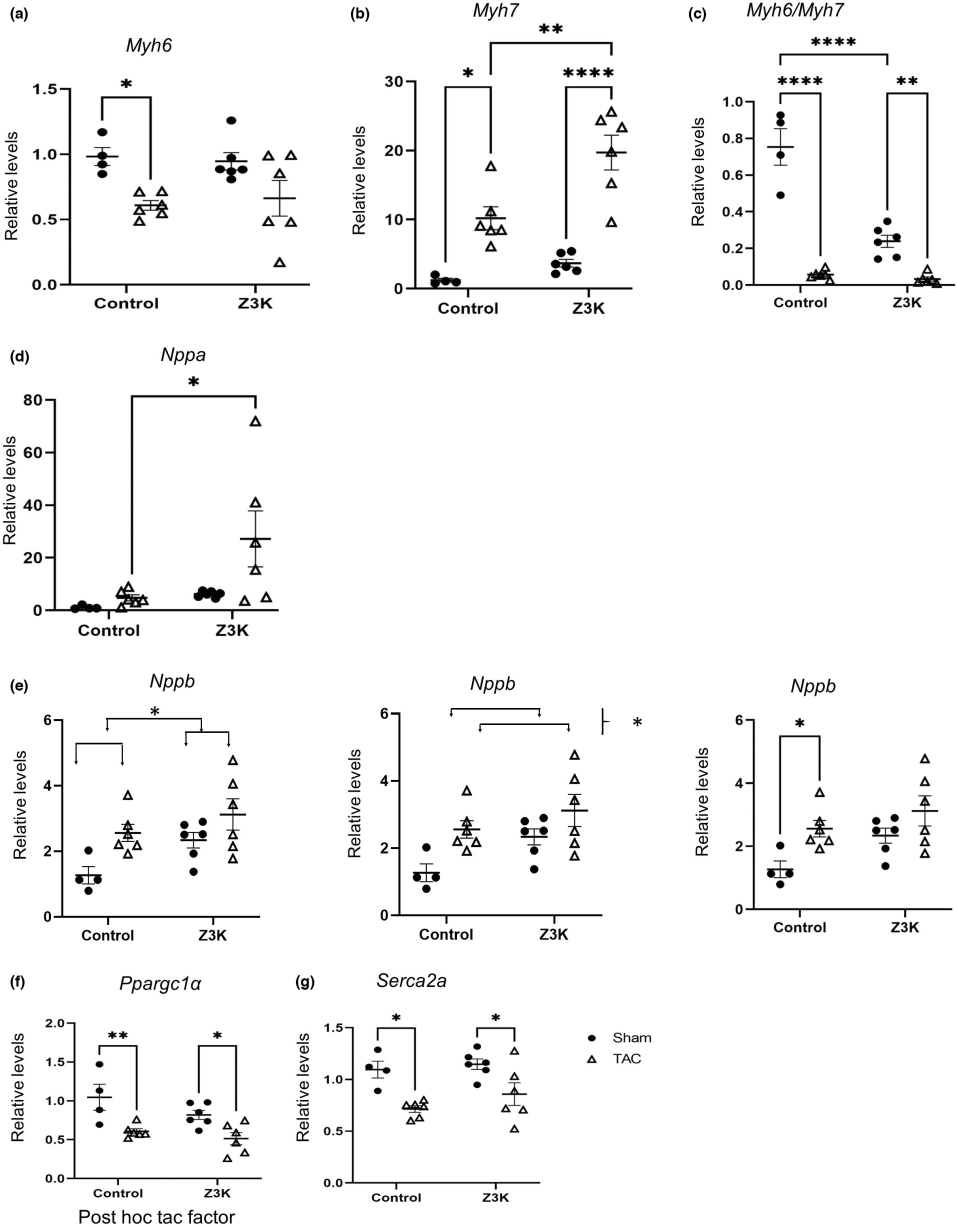
Reprogramming of genes involved in maladaptation to TAC in wildtype control and Z3K mice. We performed RT‐PCR analyses of cardiac hypertrophy related genes in control and Z3K mice treated after either sham surgery or TAC. (a) There was a decrease of *Myh6* mRNA in control mice by TAC. (b) There was an increase of *Myh7* mRNA in both genotype groups by TAC, and it was higher in Z3K after TAC compared to wildtype control after TAC. (c) *Myh6/Myh7* ratio was decreased by TAC in both genotype groups, and it was lower in Z3K sham compared to wildtype control sham. (d). There was a higher *Nppa* in Z3K mice after TAC compared to wildtype control after TAC. (e) There were both genotype difference and effect of TAC and post‐hoc showed higher *Nppb* in control mice after TAC compared to after sham. (f). There was a decrease of *Ppagc1a* in both control and Z3K mice by TAC. (g) There was a TAC effect for a decreased *Serca2a*. *p < 0.05, **p < 0.01, ***p < 0.001, ****p < 0.0001. *n* = 4–6. Two‐way ANOVA and post hoc Šidák test.

We next determined whether cardiomyocyte specific KO of ZKSCAN3 led to alterations of gene expression related to cardiac remodeling in sham or TAC mice. We found that there was a lower *Myh6* mRNA in control TAC compared to sham (Figure 4a). *Myh7* mRNA was increased by TAC in both control and Z3K mice, while the Z3K mice after TAC exhibited higher *Myh7* than control mice after TAC (Figure 4b). The *Myh6*/*Myh7* ratio was significantly decreased in both wildtype control and Z3K mice by TAC and by ZKSCAN3 knockout in sham (Figure 4c). There was a higher *Nppa* following TAC in Z3K mice but not in wildtype control mice (Figure 4d). On the other hand, *Nppb* mRNA was higher in wildtype TAC compared to sham (Figure 4e). There was a decrease of *Ppagc1a* and *Atp2a2* (a.k.a., *Serca2a*) by TAC in both control and Z3K mice (Figure 4f,g). Table 5 lists the two‐way ANOVA results of these analyses.

**TABLE 5:** Two‐way ANOVA analyses of levels of mRNAs related to cardiac hypertrophy.

	Genotype factor	TAC	Interaction
*Myh6*	ns	**	ns
*Myh7*	**	****	ns
*Myh6/myh7*	****	****	****
*Nppa*	*	ns	ns
*Nppb*	*	**	ns
*Ppagc1a*	ns	***	ns
*Serca2a*	ns	***	ns

* <0.05

** <0.01

*** <0.001

**** <0.0001.

### 3.5 The role of ZKSCAN3 in modulating the network connections between bioenergetics, structure, function and genetic remodeling of the heart

We hypothesized that there were bi‐variant relationships between parameters of cardiac function, autophagy genes, cardiac remodeling genes, and bioenergetic functions in the heart. We previously demonstrated that the networks associated with O‐GlcNAcylation enzymes and activities with mitochondrial parameters, autophagy‐related proteins as well as neurodegenerative disease‐related proteins were sex and treatment dependent (Van et al., 2022). Here we used a similar approach to reveal potential significant relationships by using Kendall bi‐variant analyses, with the following data: Body weight at sacrifice (Figure 1b); Phenotype; LV/tibia length, lung edema (WL‐DL)/tibia length, spleen/tibia length (Figure 1d–i,k,l); Cardiac function (cardiac output, EF% and FS%) (Figure 1j,m,n); Mitochondria function: citrate synthase, LDHA, mitochondrial complex I‐IV substrate‐linked activities (Figure 2); Autophagy genes (Figure 3); and cardiac remodeling related genes (Figure 4).

**FIGURE 5:**
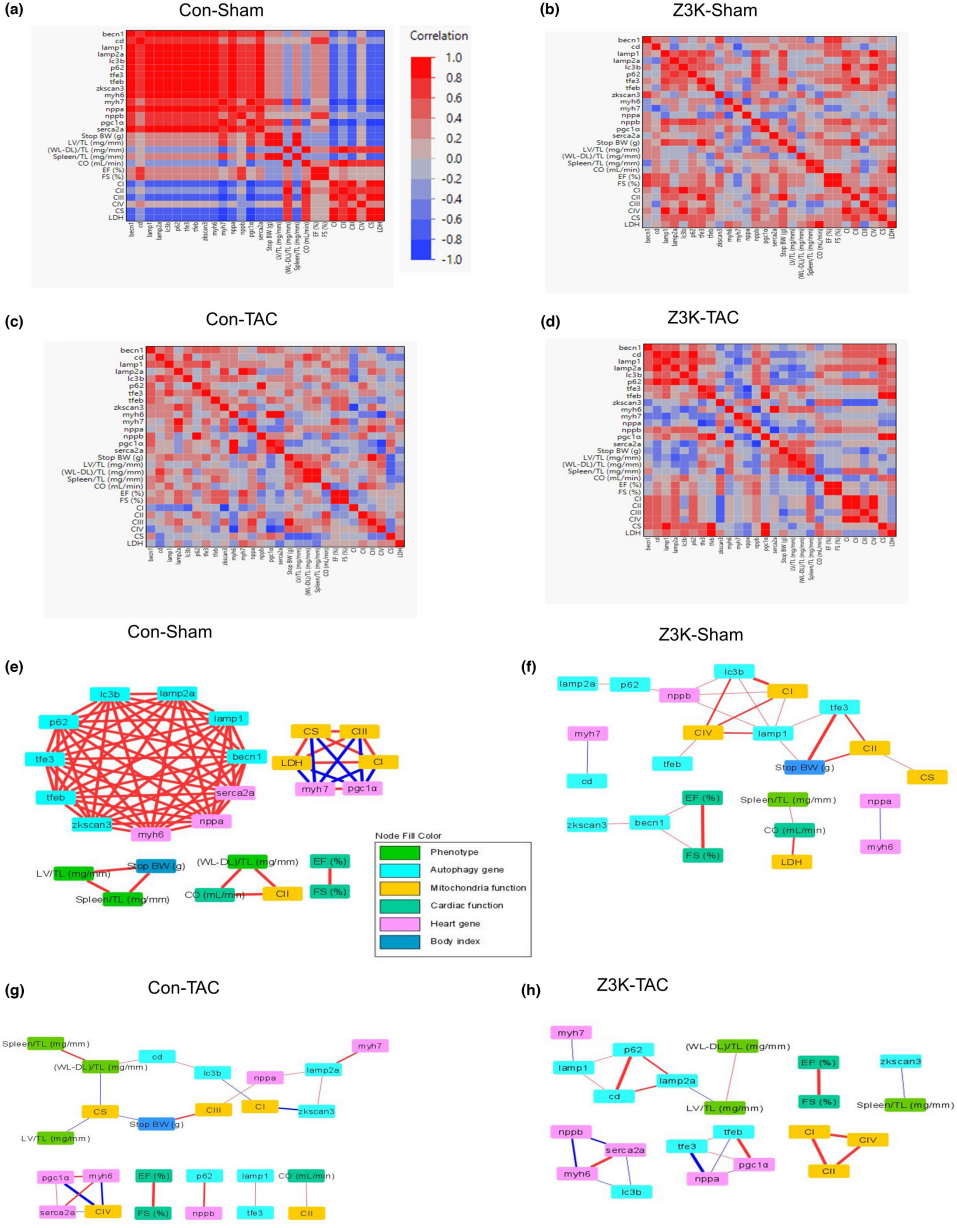
Bi‐variant analyses of relationships among body weight, cardiac parameters, autophagy and cardiac remodeling related mRNAs and mitochondrial functions. Each group of mice were analyzed separately. (a–d) Color map on Kendall's bi‐variant analyses, with positive correlation in red and negative correlation in blue. (e–h) Cytoscape diagrams of significant correlations. Shown were correlations with Kendall's Prob>|t| < 0.05. Thicker lines indicate stronger correlation. Pgc1α: *Ppargc1a*.

As shown in Figure 5a, in control sham mice, there was a significant positive correlation among both autophagy and cardiac remodeling related genes (red color), EF% was correlated with FS%, complex II, III, citrate synthase and LDH activities. However, *Ppagc1a* and *Myh7* levels were negatively correlated with complex I and III activities (blue color). The numbers of correlations were significantly decreased in Z3K mice (fewer dark red or dark blue colors) and by TAC compared to the control sham (Figure 5b,c). *Ppagc1a* is disconnected with other parameters in Z3K sham, and negatively connected with complex IV activities in wildtype control TAC (Figure 5b,c). Interestingly, Z3K mice after TAC, some of the relationships were restored; for example, the correlation between *Ctsd* with *Sqstm1/p62, between S*erca2a and *Myh6*, and between complex I and II activities; while *Ppagc1a* is positively connected to *Tfeb* and *Tfe3* is negatively connected to *Nppa* (Figure 5d). As expected, EF% is always related to FS% in all 4 groups.

We used Cytoscape to plot out all the relationships significant with Kendall's Prob>|t| < 0.05, that were derived from Figure 5a–d, with thicker lines indicating stronger relationships (Figure 5e–h). In the control sham group nearly all autophagy and cardiac remodeling mRNAs are coordinately regulated (Figure 5e). This robust network was completely abolished by ZKSCAN3 knockout, with 3 different moderately connected groups of autophagy‐cardiac remodeling genes (Figure 5f) and by TAC, with 4 different moderately connected groups of autophagy‐cardiac remodeling genes (Figure 5g). Z3K mice after TAC has also 4 different moderately connected groups of autophagy‐cardiac remodeling genes, with different members of each group compared to Z3K sham and wildtype control TAC (Figure 5h).

In addition to the major gene group, there were also 4 smaller connecting groups in wildtype control sham. *Ppagc1a* and *Myh7* levels were negatively correlated with complex I, III, citrate synthase and LDH activities. EF% was correlated with FS%. Complex II activities are correlated with cardiac output, while complex IV activities were not associated with any of the measured parameters. Body weight, LV/TL, and spleen/TL are strongly positively correlated (Figure 5e). In Z3K sham mice, the autophagy genes and the cardiac remodeling genes were segregated into 2 overlapping and 2 non‐overlapping groups. EF% and FS% are still strongly correlated, although weakly related to *Becn1* and *Zkscan3* (presumably either due to incomplete gene deletion in cardiomyocytes or due to its expression in non‐cardiomyocyte cells) (Figure 5f). Interestingly, compared to wildtype control sham (Figure 5e), mitochondrial complexes I, II, and IV activities fell into one group, with complex II and citrate synthase activities related to body weight and *Tfe*3 levels, and complex I and IV activities related to *Map1b‐lc3, Lamp1 and weakly with Tfeb, Tfe3, Nppb, Sqstm1/p62 and Lamp2a* (Figure 5f). TAC resulted in major changes in relationships between the parameters in both control and Z3K groups. In wildtype control mice with TAC, autophagy and cardiac remodeling related mRNA fell into 2 overlapping and 2 non‐overlapping groups, mitochondrial complex activities fell into 3 groups (Figure 5g). Z3K mice after TAC had a restructured relationship among mitochondrial complex I, II, and IV activities independent of other parameters, while *Tfeb* and *Tfe3* were positively related to *Ppagc1a* and negatively related to *Nppa* (Figure 5h).

## 4 DISCUSSION

Autophagy is a highly conserved process that is essential for maintaining cellular homeostasis. In the heart, impaired autophagy has been associated with cardiac dysfunction and activation of autophagy has been shown to be beneficial in the setting of ischemia/reperfusion (Hamacher‐Brady et al., 2006; Xie et al., 2011, 2014). However, those studies have primarily focused on cytosolic regulatory pathways of autophagy; however, there is increasing appreciation of the importance of transcriptional regulation of autophagy. Interestingly we found that mRNA of *Zkscan3*, a master repressor of autophagy gene expression, is decreased in response to pressure overload (TAC) in control mice. To determine whether this is an adaptive or a maladaptive response to TAC, we examined the effects of cardiomyocyte specific deletion of ZKSCAN3, in the context of pressure overload and cardiac remodeling.

Our results have demonstrated that ZKSCAN3 cardiomyocyte knockout (Z3K) mice exhibited higher mortality in response to TAC compared to wildtype control mice. We currently do not know what contributed to the mortality, and we did not collect arrhythmia data or perform post‐mortem analyses. Possible explanations include potential differences in their responses to anesthesia or TAC due to their baseline functional differences. The surviving mice after TAC exhibited cardiac hypertrophy as demonstrated by increased HW/TL, BV/TL, LV/TL, atria/TL, and pulmonary edema. Future study is needed to further determine whether similar hypertrophy (e.g., histological analyses of cardiomyocyte sizes and fibrosis) occurred in Con versus Z3K mice after TAC. However, despite these similarities in cardiac hypertrophy, the surviving Z3K mice exhibited lower body weight and higher LVPWd after TAC compared to after sham, while only control mice exhibited lower PWT%, FS%, and EF%. The observation that only Z3K mice exhibited higher LVPWd and only wildtype control mice exhibited lower PWT%, FS% and EF% in response to sham might be secondary to a slightly lower measurements in Z3K sham compared to wildtype control sham. Overall, these observations suggest that the ZKSCAN3 knockout alters the response of the heart to TAC, either due to pre‐disposition, and/or due to an increased response to TAC.

Regarding potential molecular mechanisms, we found that although TAC decreased citrate synthase activities in both wildtype control and Z3K mice, mitochondrial electron complex substrates linked respirations exhibited a wider range in Z3K mice compared to wildtype control mice, thus no significant decreases could be concluded. Regarding to gene expression, we have demonstrated that TAC in wildtype control mice suppressed transcription of *Zkscan3, Tfeb, Map1‐lc3, Ctsd, and Becn1*. It is possible that in wildtype mice, even with a decrease of *Zkscan3*, because *Tfeb* was also decreased as an adaptation to a decreased *Zkscan3*, the relative levels of nuclear TFEB:ZKSCAN3 was lower. Consequently, autophagy would not be over‐activated following decreased expression of key autophagy genes. These results indicate a dynamic coordinated transcription activation and repression of autophagy genes. Like wildtype mice after TAC, in Z3K sham mice, a decrease of *Tfeb* was observed and associated with decreases of autophagy gene expression, including *Map1‐lc3, Ctsd, and Lamp1*. These decreases of autophagy genes in Z3K sham mice may contribute to an inability to adapt to TAC with subsequent decreased mouse survival after TAC. It is likely that ZKSCAN3 is needed as a break to counteract the acceleration of autophagy by TFEB expression. Without ZKSCAN3, it would be essential to downregulated *Tfeb* as well so that the autophagy activation will not excessively stimulated.

Interestingly, *Sqstm1/p62* was higher in Z3K TAC than wildtype TAC, and *Ctsd* was higher in Z3K TAC than Z3K sham. It is possible that the increases of *Sqstm1/p62* and *Ctsd* may be due to transcription regulation independent of *Tfeb*. Both TFEB insufficiency and overexpression have been demonstrated to be detrimental to cardiac health. Its insufficiency promotes cardiac hypertrophy (Song et al., 2021), while its overexpression sensitizes the heart to chronic pressure overload (Wundersitz et al., 2022). However, in these studies, how ZKSCAN3 expression is altered have not been determined. It will be interesting to compare phenotypes of Z3K mice with mice with TFEB insufficiency and overexpression. Considering that in starved male mice, there was no difference of key autophagy genes between wildtype control and Z3K mice (Figure S7e), it is likely that sham surgery elicited significant stress and the 9 weeks after sham surgery allowed significant re‐programming of the autophagy landscape.

The decrease of *Tfeb, Map1‐lc3b, Ctsd* and *Lamp1* mRNA in sham Z3K compared to sham wildtype control (Figure 3) were surprising as ZKSCAN3 has been shown to suppression transcription of autophagy genes (Chauhan et al., 2013b). Supporting the role of ZKSCAN3 as transcription repression was the observation that *Tfeb* mRNA was increased and *Lamp1* mRNA was decreased in Z3K mice compared to wildtype mice after 24 h starvation (Figure S3d–k for mice at 4 months of age with data combining both sexes). In female mice at 2–3 months of age fed ad libitum *Becn1, Lamp1*, and *Sqstm1/P62* mRNAs were increased in Z3K mice compared to wildtype control (Figure S6b–e). There was no change of these autophagy mRNAs in males at 4 months of age after 24 h starvation (Figure S7d,e) or at 2–3 months of age fed ad libitum (Figure S8a). These differences underscore that ZKSCAN3 may have different functions in mice of different sex, age, starvation condition, and after sham or TAC surgery. Whether autophagy flux is changed due to ZKSCAN3 knockout before, shortly after, and 8 weeks after TAC is currently unknown and will be investigate in future studies.

Regarding genes related to cardiac remodeling, as expected, TAC induces significant changes of expression of *Myh6, Myh7, Myh6/Myh7 ratio, Nppb, Ppagc1a* and *Serca2a* in wildtype control mice. Most of these TAC induced changes were recapitulated in Z3K mice, especially the changes in *Ppagc1a* and *Serca2a*. *Myh6* levels in Z3K mice after TAC exhibited wider distribution. *Myh7* levels in Z3K mice after TAC was higher than in wildtype control mice after TAC. *Myh6/Myh7* ratio was lower in Z3K sham compared to wildtype control sham. *Nppa* exhibited higher and wider ranger in Z3K TAC compared to Z3K sham or wildtype control TAC. There was also a genotype effect for *Nppb* levels. Thus, these genes are both regulated in response to TAC induced cardiac remodeling and regulated either directly by ZKSCAN3‐TFEB axis or indirectly in response to a change of autophagic activities induced by ZKSCAN3 knockout. We did not use MHC‐cre as a control for TAC study. It is known that some MHC‐Cre lines exhibit a baseline defect in contractility when mice are aged. Preliminary studies in our laboratories show that the line we used did not have baseline defects at the age we performed TAC study. RNAseq experiments at 12 weeks old MHC‐Cre versus wildtype littermate control did find 4 of the genes we performed studies with shown a baseline difference, 3 were < 10%, thus unlikely the cause of the current observation of the extensive changes of gene expression in the current study. Only Nppb was 73% higher in MHC‐Cre hearts in the RNAseq study which was recapitulated in the current study (Figure 4e).

We observed TAC induced decrease of citrate synthase activities in both genotypes, consistent with a decrease in mitochondrial mass. However, there were no significant change of mitochondrial complex I‐IV substrate linked activities by ZKSCAN3 knockout or by TAC, despite a wider range of these activities in Z3K mice compared to wildtype controls. As expected, bi‐variant analyses showed that EF% is positively related to FS% in all four groups of mice. Interestingly, significant changes also occur in the landscape of relationships among phenotypes, autophagy genes, and cardiac remodeling genes. Most strikingly is the weakened relationship by ZKSCAN3 knockout and by TAC for autophagy and cardiac remodeling genes. The most likely explanation is that control sham mice have a genetic program that coordinately regulate these genes, with ZKSCAN3‐TFEB axis playing an important role in regulating autophagy related genes, and with epigenetic, transcriptional and microRNAs playing important roles in regulating cardiac remodeling genes (Akazawa & Komuro, 2003; Barry et al., 2008; Papait et al., 2020; Thum et al., 2007).

The positive relationships among mitochondrial complexes I and III activities, citrate synthase and LDH activities, and their negative relationships with *Ppagc1a* and *Myh7* mRNAs were strong in wildtype sham mice. The negative relationships with *Ppagc1a* were somewhat surprising considering that *Ppagc1a* plays a role in mitochondrial biogenesis. It is possible that mitochondrial electron transport chain activities are also regulated at the level of protein synthesis, super complex assembly, and stability. Thus, in wildtype control sham mice, the higher mitochondrial electron transport chain activities could feedback to regulate *Ppagc1a* level so that mitochondrial activities are not over‐activated. Interestingly, the control of *Ppagc1a* level was switched from complex I and III activities to complex IV activities in wildtype control mice after TAC, lost in Z3K sham and positively regulated by *Tfeb* in Z3K after TAC.

Although exhibiting a wider range, mitochondrial complex activities were generally in one cluster in Z3K sham and TAC. Complex I and IV activities were modestly related Map1‐lc3 and Lamp1, and complex II activities were modestly related to Tfe3 level, suggesting that in the absence of ZKSCAN3, mitochondrial activities are co‐dependent with autophagy gene expression. Furthermore, mitochondrial complex I, II and IV activities in Z3K TAC were not linked to citrate synthase activities. Thus, there was a dissociation between mitochondrial mass and activities in Z3K mice after TAC, potentially achieved by mechanisms targeted specific to electron transport chain proteins.

A global ZKSCAN3 knockout mouse exhibited no overt phenotype up to 8‐weeks of age (Pan et al., 2017), demonstrating that inhibiting ZKSCAN3 does not perturb normal physiology in young, unchallenged animals (Pan et al., 2017). Surprisingly, the loss of ZKSCAN3 was not associated with alterations in selected autophagy genes, or in levels of LC3II in brain, heart, or embryonic fibroblasts (Pan et al., 2017). We investigated ZKSCAN3 levels in response to bacterial infection and found that ZKSCAN3 protein is increased by exposure to *P. aeruginosa*. Furthermore, we recently generated ZKSCAN3^f/f^ mice which enabled our study on the role of ZKSCAN3 in bacterial killing in the lung (Ouyang et al., 2021). Here we have for the first time investigated cardiomyocyte specific function of ZKSCAN3. Our results have demonstrated a potentially complex role of ZKSCAN3 in mouse survival after pressure overload, a TFEB‐ZKSCAN3 coordinated regulation of autophagy in the heart, a significant change of autophagy related gene expression after TAC, a suppression of autophagy related gene expression in Z3K mice, a role of ZKSCAN3 in *Myh7, Nppa*, and *Nppb* expression in response to TAC, a reprogramming of relationship between mitochondrial electron transport chain activities with *Ppagc1a*, and autophagy and cardiac remodeling genes due to either TAC or ZKSCAN3 knockout, and a dissociation of relationship between mitochondrial electron transport chain activities with matrix citrate synthase activities. These findings will help define mechanisms of heart failure and provide new insights into therapeutic approaches.

## AUTHOR CONTRIBUTIONS

XO, SB, ZS, GAB, GH, and MSK performed experiments and data analyses. HEC performed blinded EM data analyses. SL provided guidance and technical help for EM. ARW performed TAC. MY, VDU, ARW, JCC, and JZ participated in experimental design and data interpretation. JZ wrote the manuscript. All authors edited and approved the final manuscript.

## FUNDING INFORMATION

This work was supported in part by T32HL007457 (MSK), NHLBI HL142216 (JCC, VDU, MEY, AW and JZ), P30 AG050886 (VDU, JZ) and Department of Pathology pilot grant (JCC, JZ).

## CONFLICT OF INTEREST STATEMENT

The authors declare that they have no competing interests.

## ETHICS APPROVAL AND CONSENT TO PARTICIPATE

Animal studies have been approved by UAB IACUC. No human studies.

## Supporting information


Appendix S1:

Appendix S2:
