# Supplementary material for: Cardiomyocyte ZKSCAN3 regulates remodeling following pressure‐overload
Source: Physiol Rep. 2023 May 5;11(9):e15686. doi: 10.14814/phy2.15686 (PMC10161215; doi:10.14814/phy2.15686)
Supplement: Supplementary file 1 — Appendix S1: [file PHY2-11-e15686-s001.pdf]

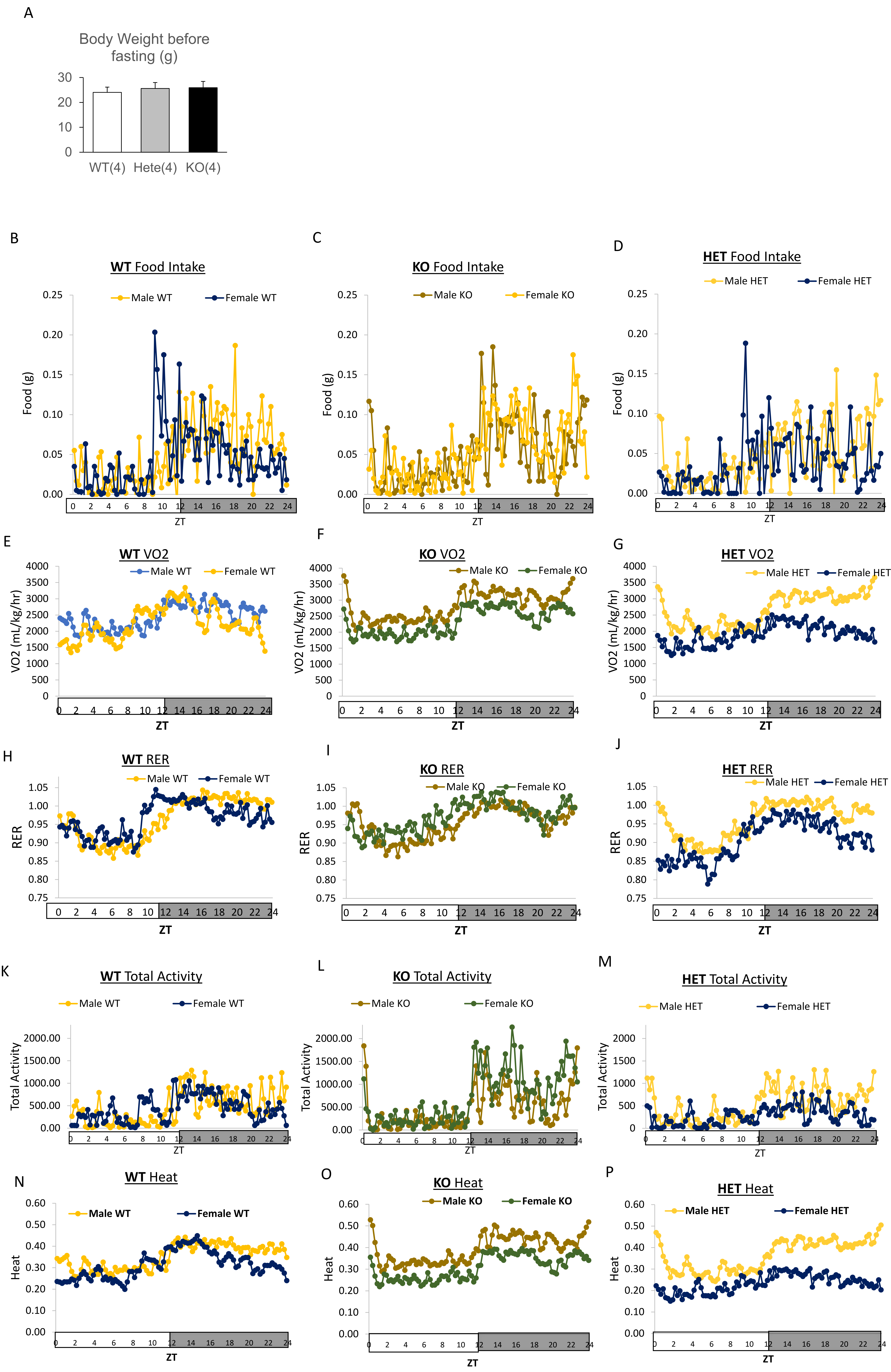

Supplemental Figure 1. Metabolic parameters in cardiomyocyte specific *Zkscan3* homozygous (KO) and heterozygotes (HET) knockout mice, compared to WT mice. (A) Wildtype, heterozygous (HET) and homozygous (KO) *Zkscan3* cardiac specific knockout mice show similar body weight at 4 months of age (2 each male and female each genotype). Wildtype, heterozygous and homozygous *Zkscan3* cardiac specific knockout (KO) mice at 4 months of age were habituated for 1 week in the CLAMS cages. Afterward, metabolic parameters were monitored for 24 hr. Food intake (**B-D**), VO2 (**E-G**), RER (**H-J**), activity (**K-M**), and energy expenditure (heat) (**N-P**), were similar for all the mice monitored. Each line represents the average of 2 mice in the same indicated group.

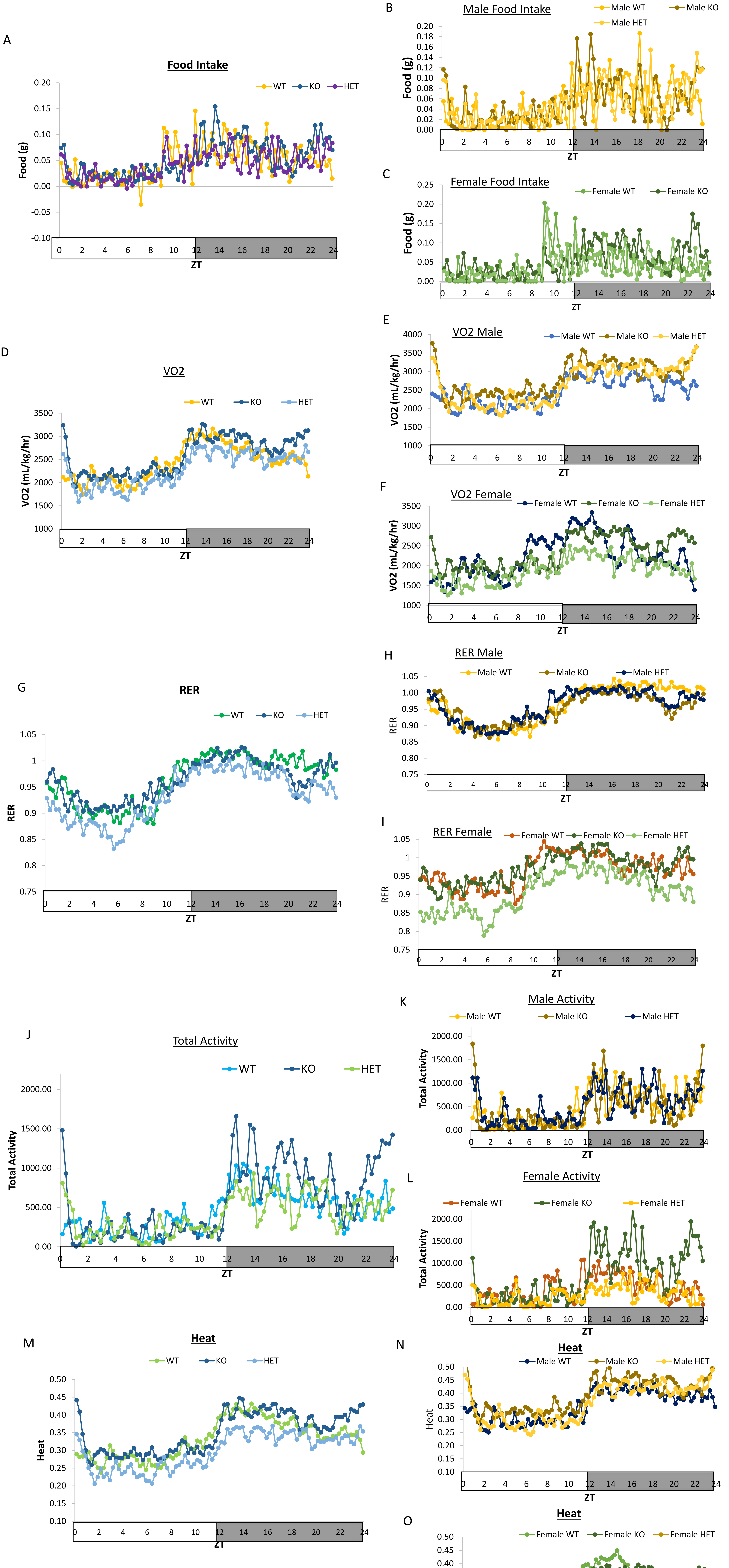

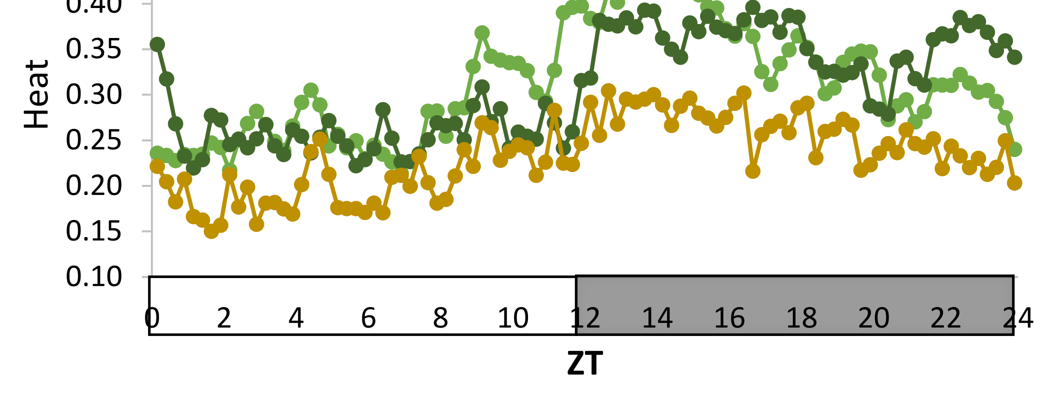

Supplemental Figure 2. Metabolic parameters in cardiomyocyte specific *Zkscan3* homozygous (KO) and heterozygotes (HET) knockout mice, compared to WT mice. A, D, G, J, M. Comparing food intake (**A**), VO2 (**D**), RER (**G**), Total activity (**J**), and Heat (**M**) among wildtype, heterozygous and homozygous *Zkscan3* knockout mice (both sexes included, all 4 months of age). Each line represents the average of 4 mice of both sexes. **B, E, H, K, N.** Comparing food intake (**B**), VO2 (**E**), RER (**H**), Total activity (**K**), and Heat (**N**) among male wildtype, heterozygous and homozygous *Zkscan3* knockout mice. Each line represents the average of 2 male mice. **C, F, I, L, O.** Comparing food intake (C), VO2 (**F**), RER (**I**), Total activity (**L**), and Heat (**O**) among female wildtype, heterozygous and homozygous Zkscan3 knockout mice. Each line represents the average of 2 female mice.

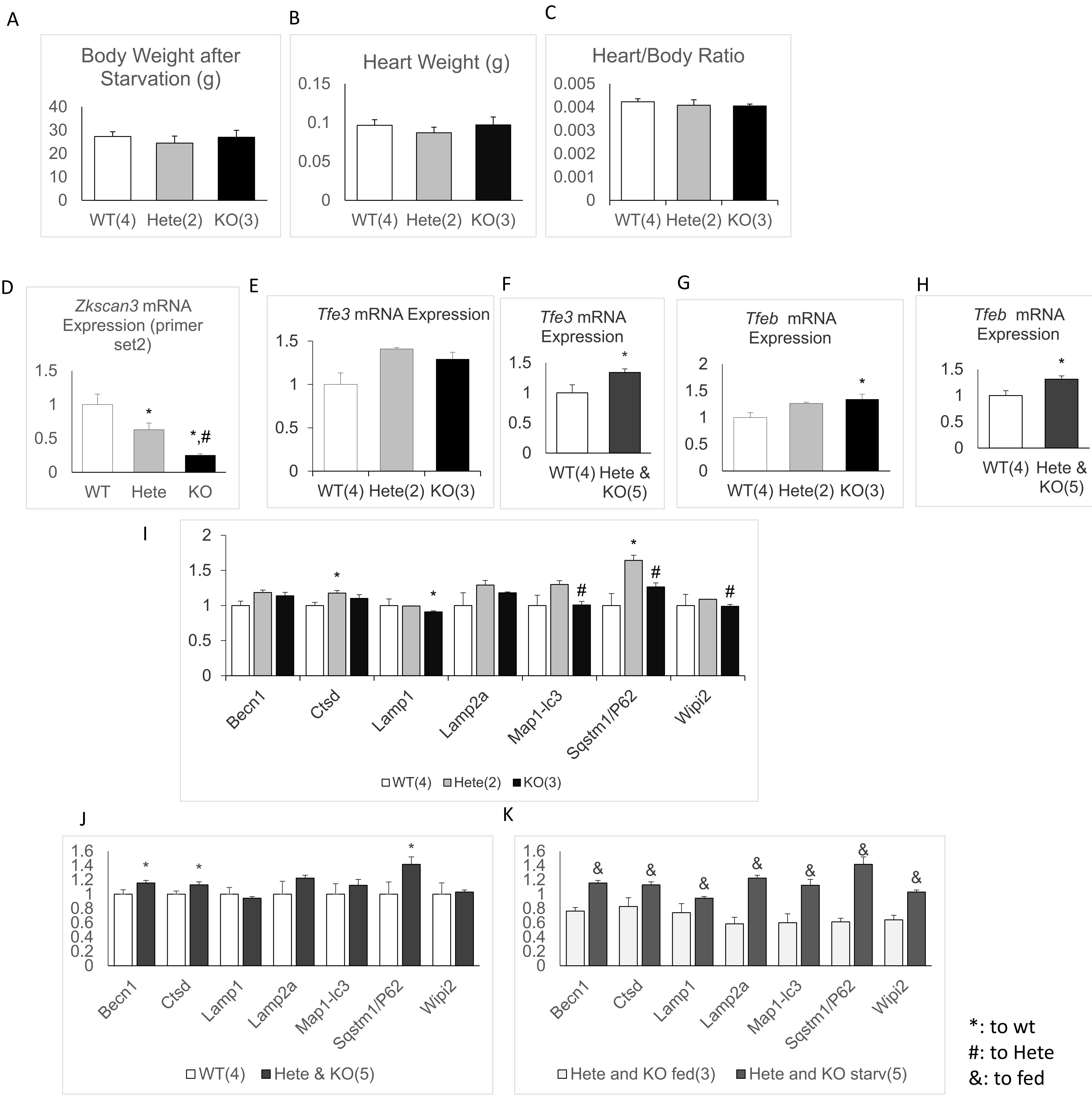

Supplemental Figure 3. Gene expression assessment in cardiomyocyte specific *Zkscan3* knock out (KO) and heterozygotes (Hete) compared to WT mice with and without fasting. After habituate for 1 week in the CLAMS cages, and 24 hr fasting, body weight **(A)**, heart weight **(B)**, and heart/body weight ratio **(C)** were similar among the 3 genotypes of mice. **D**. *Zkscan3* mRNA is downregulated in the heterozygous and homozygous knockout mice. **E-F.** There was a slight increase of *Tfe3* mRNA if combining heterozygous and homozygous mice versus the wildtype mice. **G-H.** There was an increase of *Tfeb* in homozygous knockout mice (as well as combining heterozygous and homozygous mice) compared to wildtype mice. **I**. There was a decrease of *Lamp1* in the homozygous knockout mice, and an increase of *Ctsd* and *Sqstm1/p62* in the heterozygous mice. **K.** If combine heterozygous and homozygous knockout mice to compare with wildtype mice, there was an increase of *Becn1, Ctsd, Sqstm1/p62* and *Wipi2* mRNA. **K.** If comparing fed heterozygous and knockout mice versus starved heterozygous and homozygous knockout mice, *Becn1, Ctsd, Lamp1, Lamp2a, Map1-lc3, Sqstm1/p62* and *Wipi2* mRNA were all elevated in fasting mice. *p<0.05 compared to WT, #p<0.05 compared to heterozygous (HET). &p<0.05 compared to fed. Student t-test.

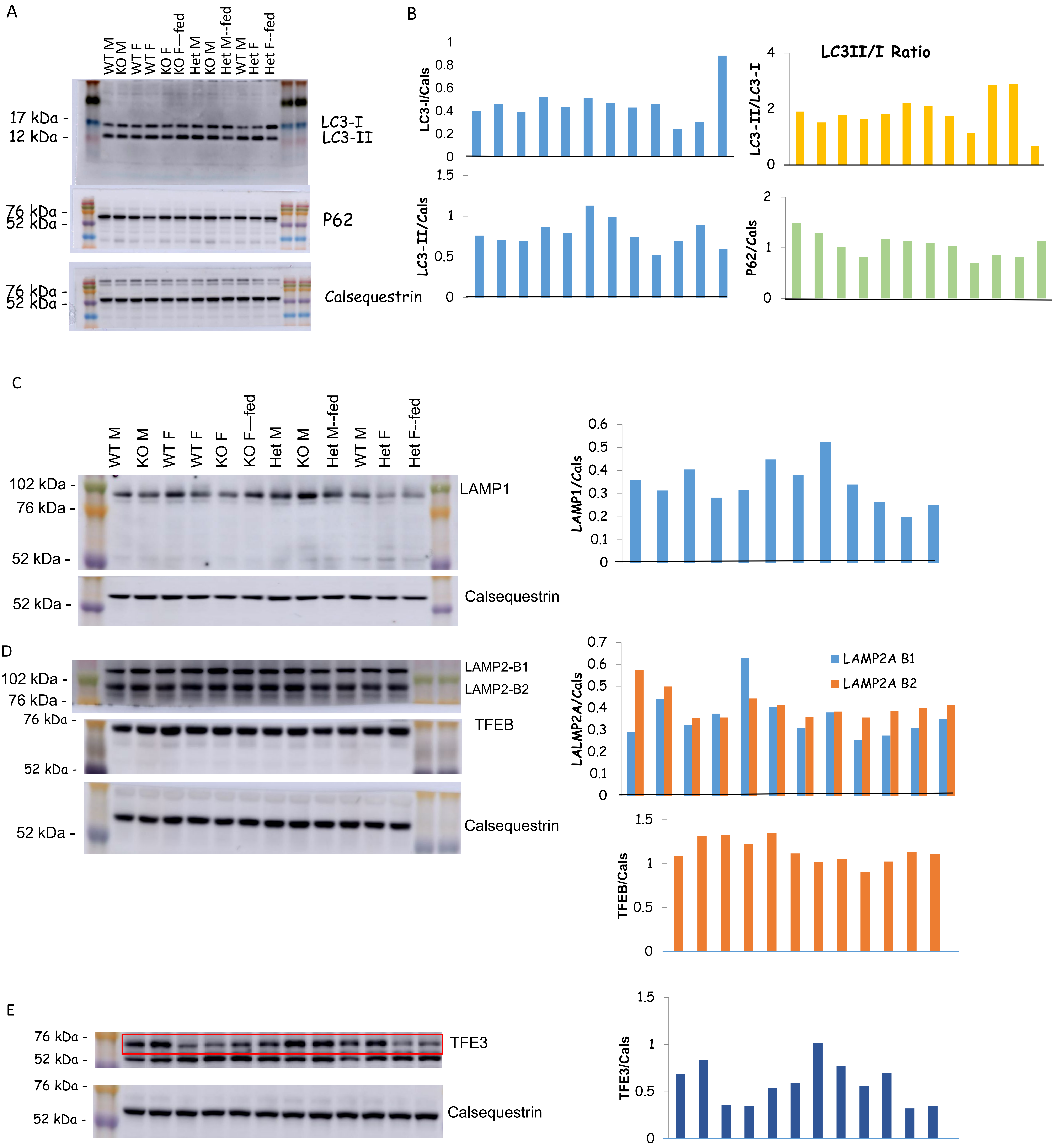

Supplemental Figure 4. No genotype dependent change in autophagy related proteins in cardiomyocyte specific *Zkscan3* knock out (KO) and heterozygotes (Het) compared to WT mice. A-B. Western blot analyses showed that the 2 fed heterozygous male (M) and female (F) mice exhibited lower LC3II/I ratio. All other mice are similar in LC3I, LC3II and LC3II/I ratio. One wildtype male and one heterozygous female had the highest LC3II/I ratio, potentially this is relative to the extent of starvation of the individual mouse. P62 (**A-B**), LAMP1 (**C**), LAMP2 and TFEB (**D**) protein levels were also similar. **E.** There was a slight increase of TFE3 protein in the heterozygous and knockout mice. Bar graph shows the quantification of each lane in sequence.

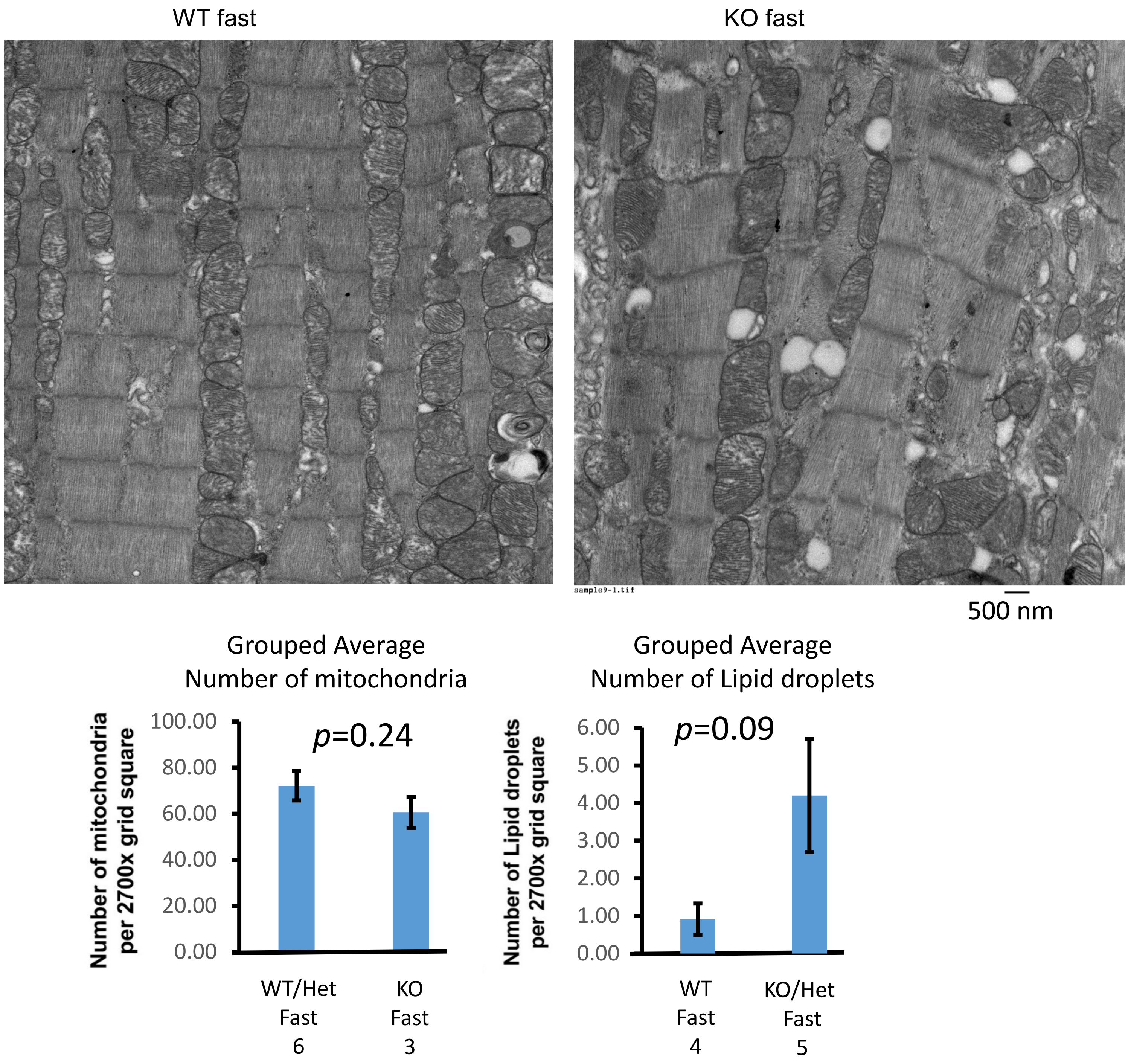

Supplemental Figure 5. Electron microscope analyses found no significant differences among the genotypes for numbers of mitochondria or numbers of lipid droplets. Shown are analyses of the fasting samples, 3 separate images each mouse were analyzed by an investigator blind to the genotype. There was a slight increase of the numbers of lipid droplet if combine heterozygous and homozygous cardiomyocyte specific ZKSCAN3 knockout mice versus wildtype mice, while the difference was not significant.

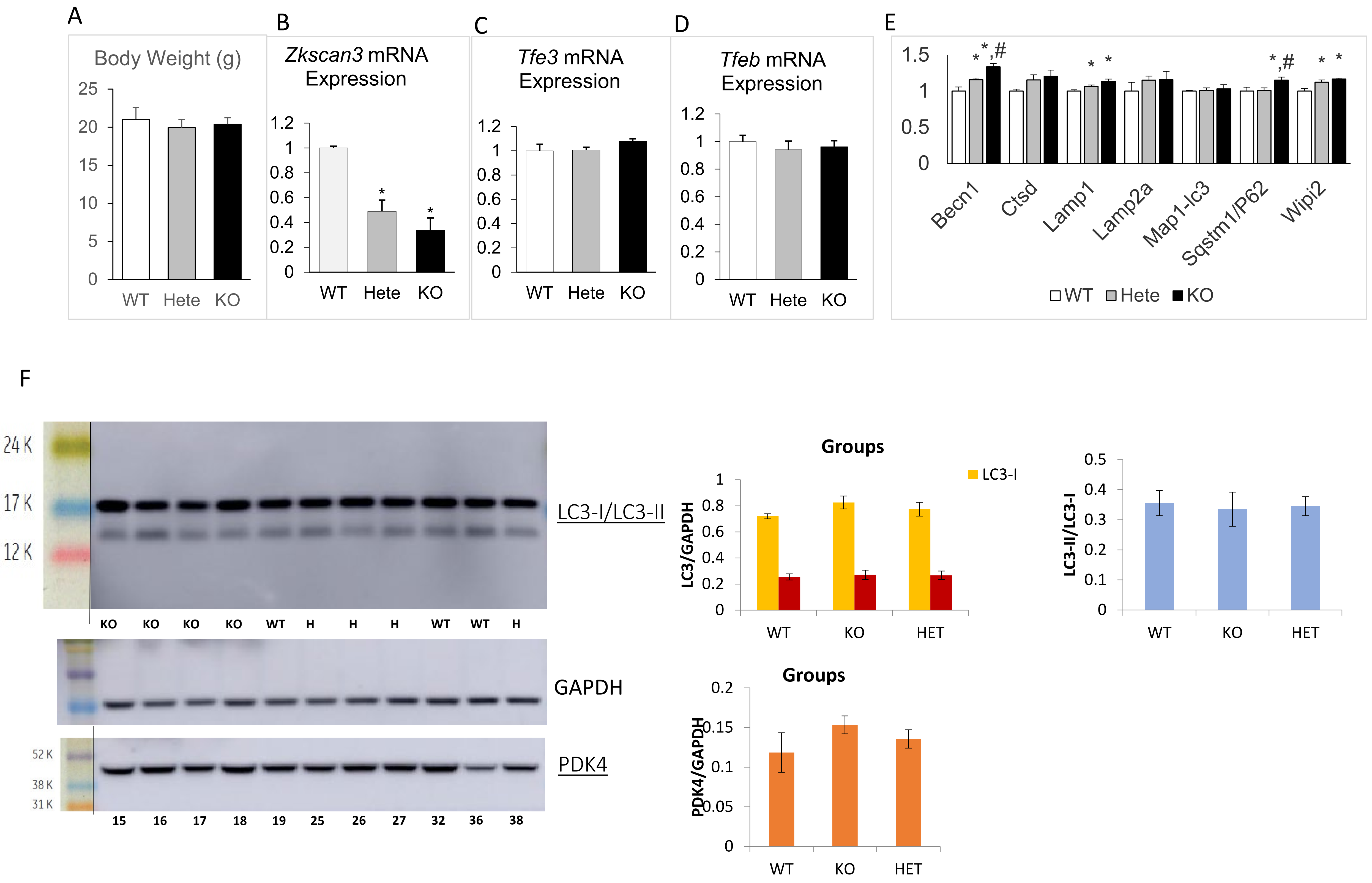

Supplemental Figure 6. No change in autophagy related proteins in cardiomyocyte specific *Zkscan3* knock out and heterozygotes compared to WT female mice fed ad libitum. All mice were female at 2-3 months of age, n=4 each wildtype, heterozygous, and homozygous. **A.** Body weight is similar among the three genotypes. **B.** *Zkscan3* mRNA is significantly decreased in heterozygous and knockouts. **C-D**. No change of *Tfe3* or *Tfeb* mRNA expression in the heterozygous or knockout mice. **E.** There was an increase of *Becn1, Lamp1,* and *Wipi2* mRNA in the heterozygous and homozygous knockout mice. There was also an increase of *Sqstm1/p62* mRNA in the homozygous knockout mice. *p<0.05 compared to WT, #p<0.05 compared to heterozygous (HET). **F.** Western blot analyses did not detect significant change of LC3I, LC3II, or PDK4 among the 3 genotypes. The lane of the molecular weight was cut and pasted for clarity.

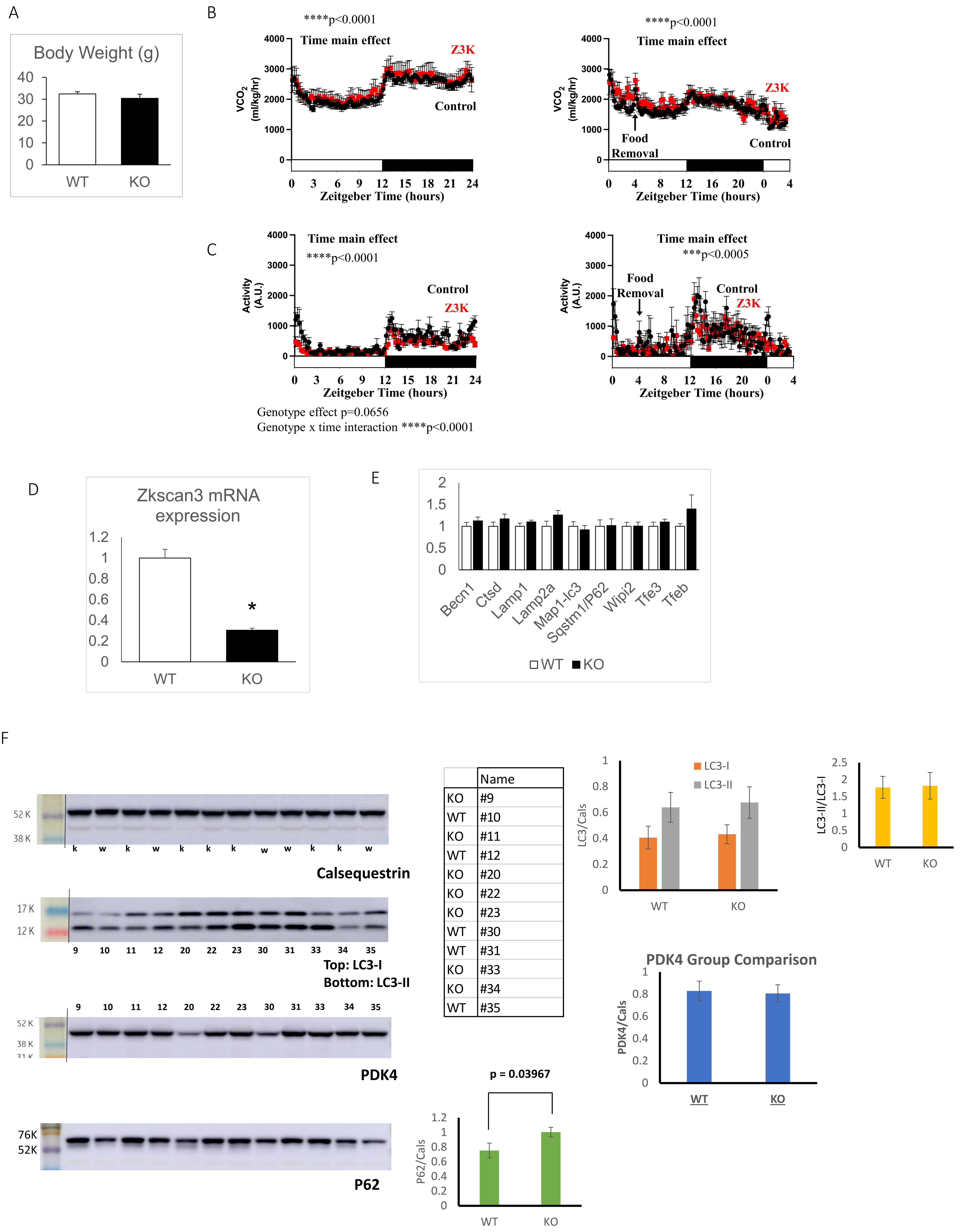

	Name
KO	#9
WT	#10
KO	#11
WT	#12
KO	#20
KO	#22
KO	#23
WT	#30
WT	#31
KO	#33
KO	#34
WT	#35

Supplemental Figure 7. Body Weight and metabolic parameters were not changed in cardiomyocyte specific *Zkscan3* knockout male mice compared to WT mice. This data are with 5 WT and 7 KO male mice at 4 months of age. **A.** Body weight before starvation was similar among the three groups. **B-C.** In the CLAMS cages for 1 week after habituation, VCO_2 and activity had time effect, but were not different between WT (n=5) and KO (n=7) mice. Activity at Fed state genotype effect p=0.0656, Genotype x time interaction ****p<0.0001 with KO mice exhibiting less activities at dark to light transition. **D.** mRNA levels of *Zkscan3* in hearts after 24 h fasting were decreased in the KO compared to the WT mice. **E.** *Becn, Ctsd (cd), Lamp1, Lamp2, Map1-lc3, Sqstm1/p62, Wipi2, Tfe3* and *Tfeb* mRNAs were similar between WT and KO. **F.** There was no difference between WT and KO with regard to LC3II or LC3II/I ratio, nor PDK4, but there was an increase of p62 protein. The lane of the molecular weight was cut and pasted for clarity.

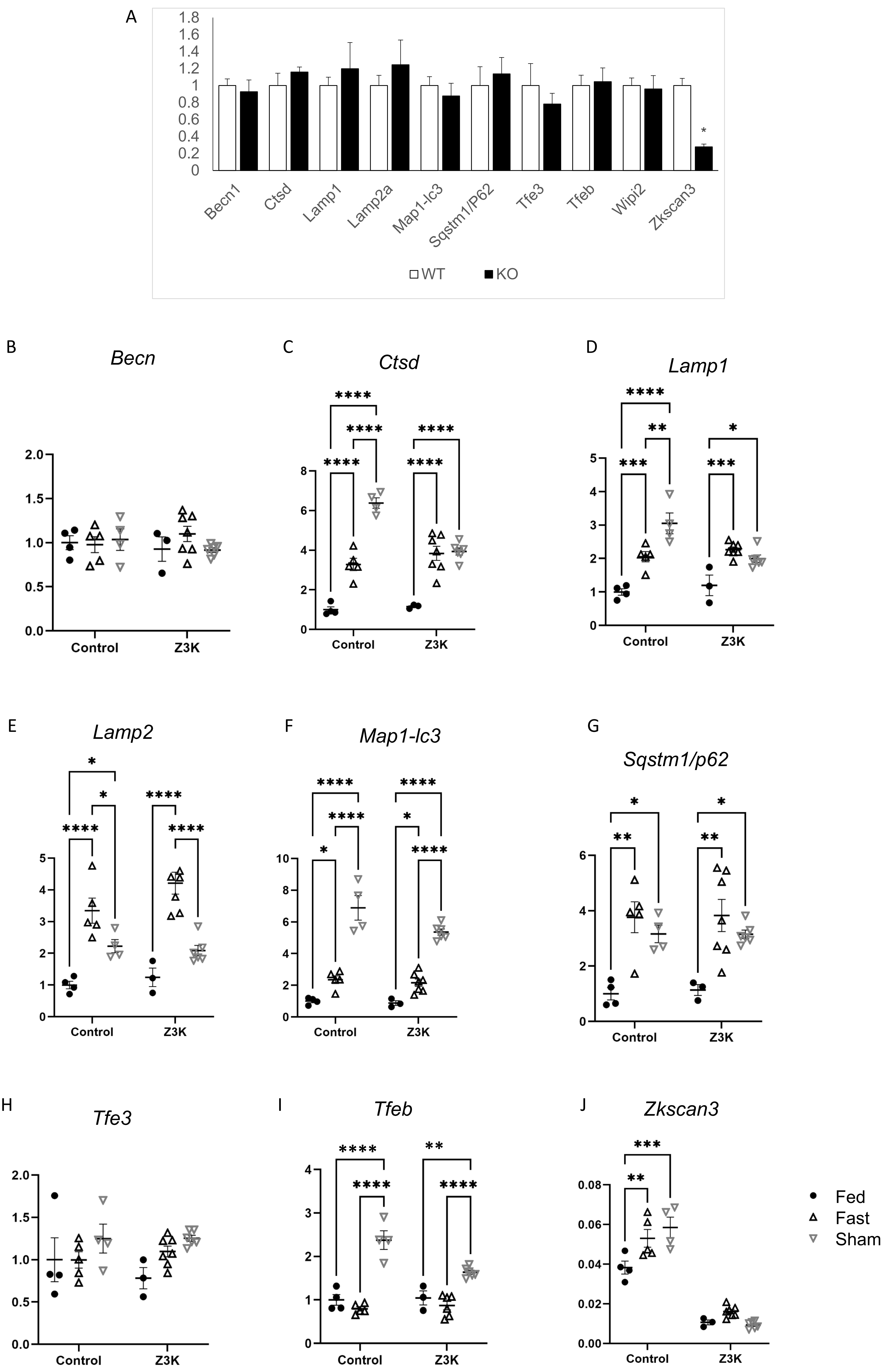

Supplemental Figure 8. Gene expression comparisons in cardiomyocyte specific ZKSCAN3 homozygous knockout male mice. A. In fed male mice, *Becn, Ctsd (cd), Lamp1, Lamp2, Map1-lc3, Sqstm1/p62, Wipi2, Tfe3* and *Tfeb* mRNAs were similar between WT and KO (male, n=4 WT and n=3 KO, 2-3 months of age). **B.** Comparison of *Becn, Ctsd (cd), Lamp1, Lamp2, Map1-lc3, Sqstm1/p62, Wipi2, Tfe3, Tfeb* and *Zkscan3* mRNAs male fed (from **Supplemental Fig.8A**), male fasted (from **Supplemental Fig.7E**, 4 months of age, n=5 WT, n=7 KO), and male undergone sham surgery (main text **Fig.2**, n=6 WT; n=6 KO, aka Z3K; sham at 10-11 weeks of age and harvest at 19-20 weeks of age). Two-way ANOVA and post hoc Tukey's comparison within each genotypes.
