# Supplementary material for: Cardiomyocyte ZKSCAN3 regulates remodeling following pressure‐overload
Source: Physiol Rep. 2023 May 5;11(9):e15686. doi: 10.14814/phy2.15686 (PMC10161215; doi:10.14814/phy2.15686)
Supplement: Supplementary file 2 — Appendix S2: [file PHY2-11-e15686-s002.docx]

**Supplemental Information**

***Zkscan3* mRNA was decreased in both heterozygotes and cardiomyocyte specific ZKSCAN3 knockout (Z3K) mice, while whole body metabolic parameters were largely unchanged.**

We generated cardiomyocyte *Zkscan3* knockout mice by breeding Zkscan3^f/f^ with MHC-cre. ZKSCAN3 whole body knockout was shown not to exhibit significant changes in mRNA levels of autophagy genes or LC3II in brain lysates or in MEFs cells with and without starvation (1). Thus we performed studies with whole body fasting. We analyzed 4 batches of mice. The first batch of mice were of 4 months of age (4 each wildtype, heterozygous and homozygous knockout, 2 each male and female). Body weight before fasting was similar among the three groups (**Supplemental Figure 1A**). We then put these mice in CLAMS cages for 1 week and monitored food intake (**Supplemental Figure 1B-D**), VO_2_ (**Supplemental Figure 1E-G**), RER (**Supplemental Figure 1H-J**), activity (**Supplemental Figure 1K-M**), and energy expenditure (heat) (**Supplemental Figure 1N-P**), and found that these were similar among different groups. Males may have higher heat/energy expenditures compared to females (statistics were not performed due to only 2 mice each group each genotype). **Supplemental Figure 2** shows the measurements comparing the three genotypes with measurements in both sexes combined or separated. Females KO appear to have higher activities compared to other two genotypes at night phase (n=2 each genotype).

Then we started fasting at ZT4 and continued CLAMS monitoring for 24 hr before sacrifice the animals. There were 2 heterozygous mice (one male and one female) and 1 knockout female that did not have food withdrawal due to the incomplete closure of the food chamber during fasting, and thus were analyzed following sacrifice as fed group. After 24 hr fasting, body weight, heart weight (including ventricles and atriums), and heart/body weight ratio were similar among the 3 genotypes of mice (**Supplemental Figure 3A-C**). Real time RT-PCR experiments showed that *Zkscan3* mRNA was downregulated in the heterozygous and homozygous knockout mice (**Supplemental Figure 3D**). When heterozygous and homozygous mice were combined, there was an increase of *Tfe3* mRNA with heterozygous and homozygous mice versus the wildtype mice (**Supplemental Figure 3E-F**). There was an increase of *Tfeb* mRNA in homozygous knockout mice compared to wildtype mice (**Supplemental Figure 3G-H**). There was an increase of *Ctsd* and *Sqstm1/p62* mRNA in heterozygous mice, a decrease of *Lamp1* mRNA in homozygous KO mice, and an increase of *Becn1*, *Ctsd* and *Sqstm1/p62* mRNA if combine both heterozygous and homozygous knockout mice to compare with wildtype mice after 24 h fasting (**Supplemental Figure 3I-J**). *Becn1, Ctsd, Sqstm1/p62, Lamp1, Lamp2, Map1-lc3,* and *Wipi2* mRNA were all elevated if comparing fed versus fasting in the combined group of heterozygous and knockout mice (**Supplemental Figure 3K**).

Western blot analyses showed that the 2 fed heterozygous male and female mice exhibited lower LC3II/I ratio. All other mice are similar in LC3I, LC3II and LC3II/I ratio, including the KO fed, this may be due to partial fed/fasting rather than the genotype (See batch 3). One wildtype male and one heterozygous female have the highest LC3II/I ratio, potentially this is relative to the extent of fasting of the individual mouse (**Supplemental Figure 4A-B**). P62 (**Supplemental Figure 4A-B**), LAMP1 (**Supplemental Figure 4C**), LAMP2 (**Supplemental Figure 4D**) protein levels are also similar. TFEB and TFE3 levels are largely unchanged (**Supplemental Figure 4D-E**). Electron microscope analyses found no significant differences among the genotypes for numbers of mitochondria or numbers of lipid droplets (**Supplemental Figure 5**).

The second batch of mice we have n=4 each wildtype, heterozygous, and homozygous female mice (2-3 months of age) without fasting. Body weight is similar among the three genotypes, while ZKSCAN3 mRNA is significantly decreased in heterozygous and knockout mice (**Supplemental Figure 6**). For this group, there was no difference in mRNA of *Tfe3* or *Tfeb*, although there is an increase of *Becn1, Lamp1, Sqstm1/p62* and *Wipi2* mRNA in the knockout mice. Western blot analyses did not detect any changes of LC3I, LC3II or PDK4. The lack of differences in LC3 may either indicate that lack of ZKSCAN3 does not alter LC3 protein level, or that this is due to that cells other than cardiomyocytes contribute significantly to the LC3 levels in the heart as detected in western blot analyses using bi-ventricular homogenates.

The third batch of mice we have 5 WT and 7 KO mice all male at 4 months of age. As the first and second batch of mice, body weight before fasting was similar among the three groups (**Supplemental Figure 7A**). We then put these male mice in CLAMS cages for 1 week and measured VCO_2_ and activities. As the first batch of mice, VCO_2_ and activity had time effect higher in the dark phase and fasting decreased the VO_2_ during dark phase in WT (n=5) and KO (n=7) mice. Interestingly, there was a genotype x time interaction (****p<0.0001) with KO mice exhibiting less activities at dark to light transition for activities at the fed state (**Supplemental Figure 7B-C**). Hearts were harvested after 24 h fasting. RT-PCR was performed and again detected decreased mRNA levels of *Zkscan3* (**Supplemental Figure 7D**), while *Becn, Ctsd, Lamp1, Lamp2, Map1-lc3, sqstm1/p62, Wipi2, Tfe3* and *Tfeb* mRNAs were similar between WT and KO (**Supplemental Figure 7E**). As prior studies indicate that fasting increases TFEB nuclear translocation and that we showed that there is an overall increase of autophagy gene expression in response to fasting (**Supplemental Figure 3K**), we expect that the function of ZKSCAN3 is diminished by fasting. There was no difference between WT and KO with regard to LC3II or LC3II/I ratio, nor PDK4, but there was an increase of p62 protein (**Supplemental Figure 7F**).

The fourth batch of mice were males of 2-3 months of age (4 WT and 3 cardiomyocyte specific ZKSCAN3 knockout mice). We found that *Becn, Ctsd, Lamp1, Lamp2, Map1-lc3, sqstm1/p62, Wipi2, Tfe3* and *Tfeb* mRNAs were similar between WT and KO (**Supplemental Figure 8A**). From these 4 batches of experiments investigating baseline phenotypes of ZKSCAN3 knockout mice, we found ZKSCAN3 knockout did not result in changes in general metabolic activities (CLAMS). Autophagy gene expression is still induced in ZKSCAN3 HET and KO heart. There were increased mRNA expression of some of the autophagy genes (becn1, p62, lamp1, and wipi2) in KO mice without fasting. There was also a modest increase of p62 protein in male knockout mice under fasting condition.

Then, we compared autophagy gene expression with male mice fed at 2-3 months of age (**Supplemental Figure S8A**) with male mice fasted at 4 months of age (**Supplemental Figure S7D and E**), and underwent sham surgery at nearly 5 months of age (**Figure 2**). We found that there were no differences of *Becn* **Supplemental Figure S8B**) or *Tfe3* mRNA (**Supplemental Figure S8H**). Interestingly, in wildtype control mice, *Tfeb* mRNA was higher in the sham surgery group (**Supplemental Figure S8I**). *Zkscan3* and *Sqstm1/p62* mRNA were higher in fasted and sham surgery group (**Supplemental Figure S8J**). *Ctsd, Lamp1,* and *Map1-lc3* were higher in wildtype control fasted group compared to fed, and even higher in sham group compared to fasting (**Supplemental Figure S8C, D, and F**).  *Lamp2* mRNA was higher in sham group compared to fed, and even higher in fasting group compared to sham (**Supplemental Figure S8E**). In Z3K mice, *Tfeb, Sqstm1/p62, Lamp2,* and *Map1-lc3* exhibited similar trend as wildtype control mice with relative levels among the fed, fasting and sham groups, while *Ctsd* and *Lamp1* were no longer higher in sham group compared to fasting group. These differences may be due to combined effect of age and fasting or sham surgery, but nonetheless underscore that ZKSCAN3 and TFEB were regulated differently, and that their combined levels contribute to the downstream transcription programs.

**Supplemental Method:**

**CLAMS** (comprehensive laboratory animal monitoring system) study**:** Animals were kept on a 12h/12h light/dark cycle and single housed in the CLAMS cages for a week before either fed or fast (food withdrawal) at ZT6, followed by the measurement for 24 hrs. Data were binned in 15 min increments. Data = mean ± SEM. ZT: Zeitgeber time. Volume of O_2_ inhaled, food consumption, energy expenditure (indirect calorimetry), total activity (total number of beam breaks), and RER (respiratory exchange ratio used to estimate the relative reliance for carbohydrate versus fatty acid metabolism, and aerobic versus anaerobic metabolism; a value of 0.7 represents almost complete reliance on fatty acid metabolism, a value of 1.0 represents reliance on aerobic carbohydrate metabolism, and >1.0 represents reliance on aerobic carbohydrate metabolism) were measured using a comprehensive laboratory animal monitoring system (CLAMS; Columbus Instruments Inc.) in a 12 h/12 h light/dark cycle controlled room for 24 hrs (2).

**Transmission electron microscopy:** A small middle segment of the ventricles were fixed with 2% glutaraldehyde in 0.1 M sodium cacodylate buffer, followed by 2% osmium tetroxide in 0.1 M sodium cacodylate buffer, dehydrated and embedded in Epon. Transmission electron microscopy was performed were acquired at the UAB high-resolution imaging facility (3-5). Mitochondria and lipid number counting by an investigator blinded for the genotype.

**Western Blot Analysis:** Bi-ventricular tissues were prepared in RIPA buffer supplemented with protease/phosphatase inhibitor cocktail (Thermofisher cat #1861284).  The DC Lowry (Bio-Rad) assay was used to determine protein quantification and 10-20 µg protein per lane were loaded onto 10 or 12% PAGE SDS gels. After separation, proteins were transferred to PVDF or nitrocellulose membrane to probe by the following antibodies: p62 (Abnova, H00008878-M01), Microtubule-associated protein 1 light chain 3 alpha/LC3 (Sigma, L8918), LAMP1 (Ab25245; Abcam Cambridge, MA), TFE3 (HPA023881), TFEB (A303-673A; Bethyl, Montgomery, TX), Calsequestrin (Abcam ab3516), β-actin (Sigma, A5441), and GAPDH (Millipore, MAB374). Each experiment was carried out from heart homogenates obtained from indicated groups of mice.

1. Pan H, Yan Y, Liu C, and Finkel T. The role of ZKSCAN3 in the transcriptional regulation of autophagy. *Autophagy.* 2017;13(7):1235-8.

2. Ouyang X, Ahmad I, Johnson MS, Redmann M, Craver J, Wani WY, et al. Nuclear receptor binding factor 2 (NRBF2) is required for learning and memory. *Lab Invest.* 2020;100(9):1238-51.

3. Collins H, Kane M, Litovsky S, Darley-Usmar V, Young M, Chatham J, et al. Mitochondrial morphology and mitophagy in heart diseases: qualitative and quantitative analyses using transmission electron microscopy (TEM). *Frontier in Aging.* 2021;1.

4. Collins HE, He L, Zou L, Qu J, Zhou L, Litovsky SH, et al. Stromal interaction molecule 1 is essential for normal cardiac homeostasis through modulation of ER and mitochondrial function. *Am J Physiol Heart Circ Physiol.* 2014;306(8):H1231-9.

5. Collins HE, Pat BM, Zou L, Litovsky SH, Wende AR, Young ME, et al. Novel role of the ER/SR Ca(2+) sensor STIM1 in the regulation of cardiac metabolism. *Am J Physiol Heart Circ Physiol.* 2019;316(5):H1014-H26.
